# Whole genome sequence analysis of population structure and insecticide resistance markers in *Anopheles melas* from the Bijagós Archipelago, Guinea-Bissau

**DOI:** 10.1186/s13071-024-06476-2

**Published:** 2024-09-18

**Authors:** Sophie Moss, Elizabeth Pretorius, Sainey Ceesay, Eunice Teixeira da Silva, Harry Hutchins, Mamadou Ousmane Ndiath, Holly Acford-Palmer, Emma L. Collins, Matthew Higgins, Jody Phelan, Robert T. Jones, Hristina Vasileva, Amabelia Rodrigues, Sanjeev Krishna, Taane G. Clark, Anna Last, Susana Campino

**Affiliations:** 1 Faculty of Infectious and Tropical Diseases, London School of Hygiene & Tropical Medicine, London, WC1E 7HT UK; 2 Medical Research Council Unit The Gambia at the London School of Hygiene and Tropical Medicine, Banjul, The Gambia; 3 Projecto de Saúde Bandim, Bissau, Guinea-Bissau; 4 Ministério de Saúde Pública, Bissau, Guinea-Bissau; 5 Clinical Academic Group, Institute for Infection and Immunity, and St. George’s University Hospitals NHS Foundation Trust, St. George’s University of London, London, UK; 6 Centre de Recherches Médicales de Lambaréné (CERMEL), Lambaréné, Gabon; 7 Institut Für Tropenmedizin Universitätsklinikum Tübingen, Tübingen, Germany; 8 Faculty of Epidemiology and Population Health, London School of Hygiene & Tropical Medicine, London, WC1E 7HT UK

**Keywords:** Anopheles melas, Whole-genome sequencing, Guinea-Bissau, Population structure, Insecticide resistance, Malaria

## Abstract

**Background:**

*Anopheles melas* is an understudied malaria vector with a potential role in malaria transmission on the Bijagós Archipelago of Guinea-Bissau. This study presents the first whole-genome sequencing and population genetic analysis for this species from the Bijagós. To our knowledge, this also represents the largest population genetic analysis using WGS data from non-pooled *An. melas* mosquitoes.

**Methods:**

WGS was conducted for 30 individual *An. melas* collected during the peak malaria transmission season in 2019 from six different islands on the Bijagós Archipelago. Bioinformatics tools were used to investigate the population structure and prevalence of insecticide resistance markers in this mosquito population.

**Results:**

Insecticide resistance mutations associated with pyrethroid resistance in *Anopheles gambiae* s.s. from the Bijagós were absent in the *An. melas* population, and no signatures of selective sweeps were identified in insecticide resistance-associated genes. Analysis of structural variants identified a large duplication encompassing the cytochrome-P450 gene *cyp9k1*. Phylogenetic analysis using publicly available mitochondrial genomes indicated that *An. melas* from the Bijagós split into two phylogenetic groups because of differentiation on the mitochondrial genome attributed to the cytochrome C oxidase subunits COX I and COX II and the NADH dehydrogenase subunits 1, 4, 4L and 5.

**Conclusions:**

This study identified an absence of insecticide-resistant SNPs common to *An. gambiae* in the *An. melas* population, but did identify structural variation over insecticide resistance-associated genes. Furthermore, this study presents novel insights into the population structure of this malaria vector using WGS analysis. Additional studies are required to further understand the role of this vector in malaria transmission.

**Graphical abstract:**

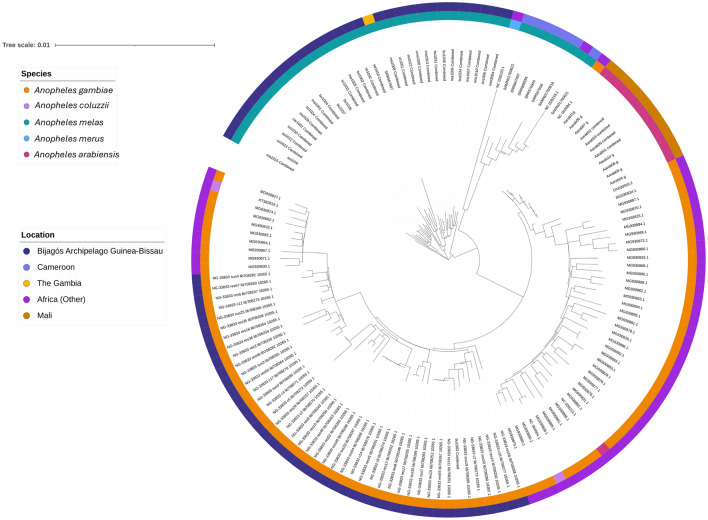

**Supplementary Information:**

The online version contains supplementary material available at 10.1186/s13071-024-06476-2.

## Background

Malaria is a persistent public health problem in Guinea-Bissau, West Africa, where the population of 2.1 million people experienced an estimated 225,200 malaria cases and 1023 malaria deaths in 2022 [1]. The Bijagós Archipelago (Bijagós) is a group of 88 islands and islets located approximately 50 km off the coast of Guinea-Bissau and is a designated UNESCO Biosphere Reserve [2]. The Archipelago is home to approximately 25,000 people, who live on 19 permanently inhabited islands [3]. Malaria transmission on the Bijagós is highly seasonal and stable [4], and *Plasmodium falciparum* prevalence on the Archipelago can peak at up to 15% at the end of the rainy season in November [5].

A survey in 2017 on Bubaque Island, the most populous island of the Archipelago, identified all *Anopheles* species on Bubaque to be members of the *Anopheles gambiae *sensu lato (s.l.) complex. Of the species present, *Anopheles gambiae *sensu stricto (s.s.) was identified as the primary malaria vector and *Anopheles melas* as having a role in low level transmission during the dry season [4]. *Anopheles melas* is a saltwater-tolerant species able to sustain population numbers during the dry season by laying eggs in brackish water, giving this species a particular advantage during the dry season when freshwater oviposition sites have dried up [6]. The Bijagós Archipelago has an abundance of mangroves and mud flats [2], which are commonly associated with the presence of *An. melas* [7, 8]. A larger survey conducted on 16 of the inhabited islands of the Archipelago was conducted between October and December 2019 during the peak malaria transmission season. This survey used indoor and outdoor light-traps and identified 85.2% of trapped mosquitoes to be *An. melas* (Pretorius et al. 2024, in review). A sub-sample of mosquitoes were investigated for *Plasmodium falciparum* sporozoite positivity using circumsporozoite protein (CSP) ELISA, which calculated a sporozoite rate of 0.86% and identified all CSP-positive specimens collected as *An. melas* (Pretorius et al. 2024, in review). This study indicates that *An. melas* may be important in malaria transmission on the Bijagós, particularly regarding residual transmission during the dry season (Pretorius et al. 2024, in review). This is supported by previous studies which have identified *An. melas* to have a role in malaria transmission, including in Senegal [9], The Gambia [10, 11] and Equatorial Guinea [12].

Vector control on the Bijagós relies on the use of insecticide-treated nets (ITNs) impregnated with pyrethroid insecticides, which are distributed every 3 years and have high estimated coverage and usage of around 90% [13, 14]. Pyrethroid ITNs are the most successful vector control intervention developed to date, having prevented approximately 68% of malaria deaths in Africa between 2000 and 2015 [15]. Alarmingly, resistance to pyrethroids is highly prevalent worldwide [16]. Of all countries that reported resistance data to WHO between 2010 and 2020, 87% declared pyrethroid resistance in at least one malaria vector [1]. Resistance to pyrethroids has been associated with single-nucleotide polymorphisms (SNPs) in the voltage-gated sodium channel gene (*vgsc*) of *An. gambiae*, including L995F and L995S, also known as the *kdr* west and *kdr* east alleles [17, 18], and the N1570Y mutation [19]. In addition, target site mutations in the *gste2* gene have been associated with pyrethroid resistance, including L119V and I114T [20]. Resistance to pyrethroids has been associated with copy number variants (CNVs) encompassing genes in three major enzyme families: cytochrome-P450s, esterases and glutathione-S-transferases (GSTs) [21–24].

Insecticide resistance is a growing threat to the control of malaria. This threat has propelled the understanding of *Anopheles* genetic variation through international collaborations such as the Anopheles Gambiae 1000 Genomes Project [25]. Genomics research has focused on the key malaria vectors *An. gambiae* s.s. and *Anopheles coluzzii*, but few studies have investigated *An. melas*. On the Bijagós, a previous study on Bubaque Island investigated the presence of the *kdr* east and west alleles in *An. gambiae* s.l. mosquitoes using targeted PCR sequencing [4], and a subsequent study across 13 islands investigated the prevalence of known insecticide resistance mutations using high-throughput multiplex-amplicon sequencing [26]. This study identified four mutations associated with insecticide resistance in *An. melas* at low prevalence. This included three mutations in the *vgsc* gene, L995F, N1570Y and A1746S, one mutation in the *rdl* gene, A296G, and no known insecticide resistance mutations in the *ace1* or *gste2* genes [26]. However, no previous studies using whole-genome sequence (WGS) data from *An. melas* on the Bijagós Archipelago have been conducted, and population structure and signatures of selection have not previously been investigated. Furthermore, to our knowledge, only two studies analysing WGS data from *An. melas* have previously been published. This includes a study of Pool-seq WGS data from *An. melas* in The Gambia, Cameroon and Equatorial Guinea, where the DNA from several individual mosquitoes was pooled prior to sequencing [27] and the Anopheles 16 Genomes Project, which produced the reference genome assembly for *An. melas* [28]. The previous study using Pool-seq WGS data identified three genetically distinct clusters of *An. melas* on the West African coast, *An. melas* West, *An. melas* South and *An. melas* Bioko, which ranged respectively from The Gambia to Northwest Cameroon, Southeast Cameroon to Angola and Bioko Island in Equatorial Guinea [27, 29]. Genetic differentiation between these clusters was high and mostly distributed evenly across the genome, with elevated differentiation along the X chromosome, indicative of allopatric divergence [27].

Here, we generate and analyse WGS data from 30 individual mosquitoes of this little-known species, collected during the 2019 vector survey on the Bijagós Archipelago, combined with WGS data from seven *An. melas* specimens made available through the *Anopheles* 16 Genomes Project [28]. WGS data are a valuable resource which we utilise to investigate the genetic diversity of this mosquito population, the presence of SNPs associated with insecticide resistance and genomic signatures of selection in this species.

## Methods

### Mosquito sampling

**Fig. 1:**
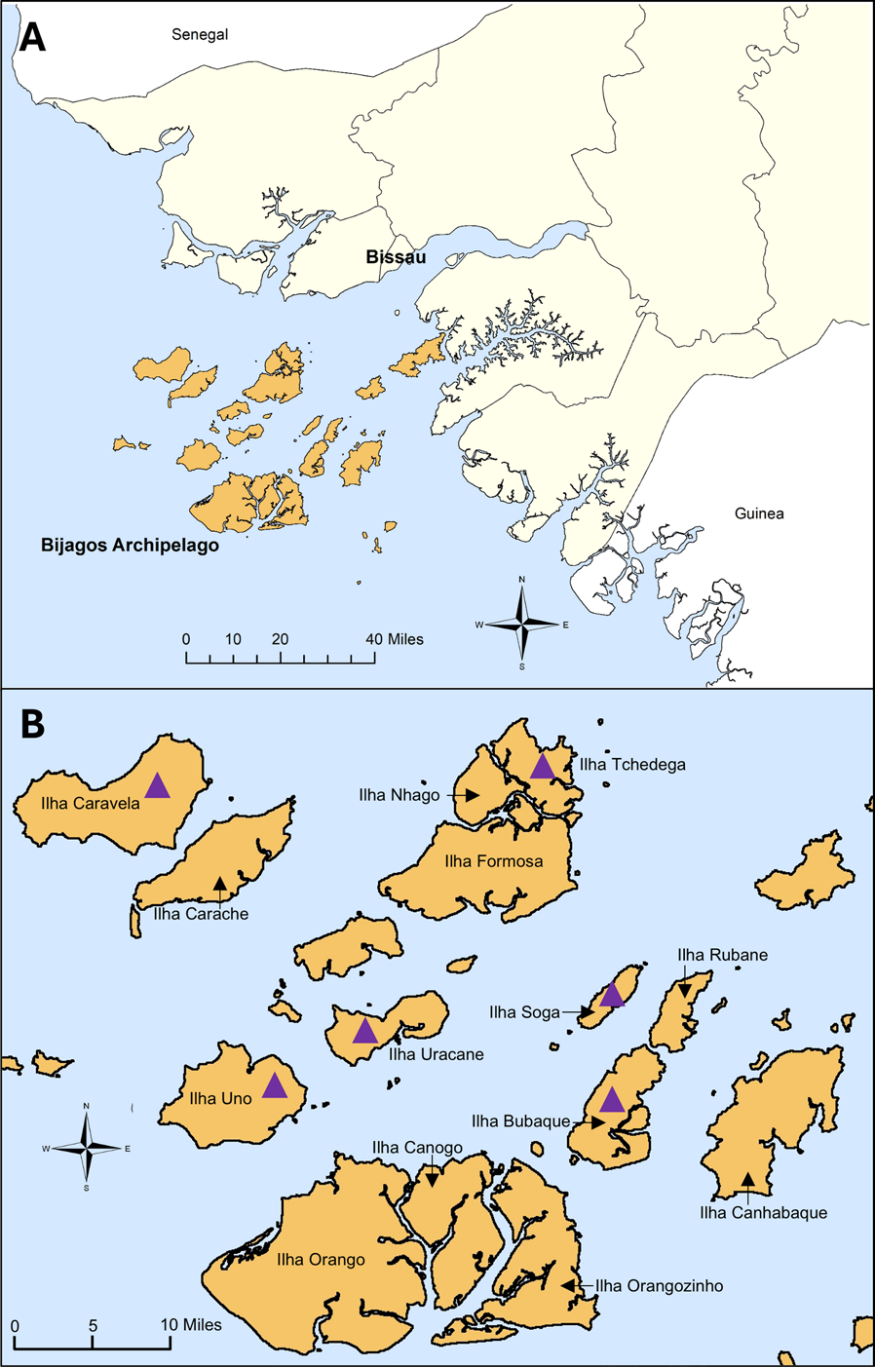
Mosquito sample collection sites. **A** Location of Bijagós Archipelago, created using ArcGIS ArcMap 10.8.1. **B** Mosquitoes were collected from the six islands labelled with purple triangles. Map from OpenStreetMap 2023‑05‑06

Mosquitoes were collected from the Bijagós Archipelago during October and November 2019 using CDC indoor and outdoor light traps (Model 512; John W. Hock Co., Gainesville, FL, USA) using previously described methodology [30]. This included mosquitoes from six different islands across the Archipelago highlighted with purple triangles in Fig. 1: Soga, Bubaque, Tchedega (Maio), Uno, Caravela and Uracane. Collected mosquitoes were separated by genus, and *Anopheles* mosquitoes were morphologically identified using previously described keys [31]. All mosquitoes were identified as belonging to the *An. gambiae* s.l. complex.

### DNA extraction

DNA extraction was conducted following the manufacturer’s instructions, using the QIAamp^®^ 96 DNA QIAcube^®^ HT Kit (Qiagen) with the QIAcube Extraction Robot. DNA was eluted in 80 µl AE buffer and stored at – 20 ℃. No additional processing of DNA, for example whole-genome amplification or selective genome amplification, was conducted prior to sequencing.

### Species identification

Female mosquitoes were then identified to species at the Medical Research Council Unit The Gambia at London School of Hygiene & Tropical Medicine using PCR-RFLP to distinguish between members of the *An. gambiae* s.l. complex, based on the protocol of Fanello et al. [32]. This used primers to amplify the intergenic spacer (IGS) region, which differs in size between members of the *An. gambiae* complex. The following primers were used: Universal F: GTGTGCCCCTTCCTCGATGT; *Anopheles gambiae* R: CTGGTTTGGTCGGCACGTTT; *An. arabiensis* R: AAGTGTCCTTCTCCATCCTA; *An. melas* R: TGACCAACCCACTCCCTTGA. Amplified PCR products were then digested using the HhaI enzyme to differentiate *An. gambiae* s.s. and *An. coluzzii* specimens. The band sizes of the PCR products were visualised using electrophoresis with the QIAxcel capillary electrophoresis system (Qiagen). The band sizes of PCR product were analysed to distinguish species: *An. gambiae* s.s. (257 and 110 bp), *An. arabiensis* (292 bp), *An. melas* (435 bp), *An. coluzzii* (367 bp) and *An. coluzzii/An. gambiae* s.s. hybrid (257 bp, 110 bp and 367 bp).

### Whole-genome sequencing and bioinformatic analysis

The DNA of 30 *An. melas* mosquitoes from six different islands across the archipelago was whole-genome sequenced at Eurofins Genomics using the Illumina Novaseq 6000 (2 × 150 bp configuration). This included 11 *An. melas* from Soga, 7 from Bubaque, 7 from Tchedega (Maio), 3 from Uno, 1 from Caravela and 1 from Uracane. Average read depth for all samples across the whole genome was 32.61. Over 55% of the genome for each sample had a read depth ≥ 20. Publicly available *An. melas* data were included for population analyses. This included one sample from The Gambia and six samples from Cameroon, made available through the Anopheles 16 Genomes Project [28, 33]. Mosquitoes were phenotypically identified as female during sampling, and this was called again using the modal coverage ratio between chromosome 3R and chromosome X (with a ratio between 0.4 and 0.6 = male and ratio between 0.8 and 1.2 = female, with other ratios leading to sample exclusion, as previously described [34]).

Raw WGS data were trimmed using *trimmomatic* (version 0.39) [35] before aligning to the *Anopheles gambiae* (AgamP4) reference genome and the *Anopheles melas* (AmelC2) reference genome, using *bwa-mem* software (default parameters)[36]. The AgamP4 alignment was taken forward as the AgamP4 reference genome is of better quality than the *An. melas* reference genome, and the percentage of reads which mapped to AgamP4 was higher (average 92.8%) than the percentage of reads which mapped to AmelC2 (average 79.3%). Furthermore, the AmelC2 reference genome is not a chromosomal level assembly, consisting of over 22,000 scaffolds with a mean N50 value of 18,103, compared to a AgamP4, which is a chromosomal level assembly with mean N50 value of 49,364,325. Therefore, mapping of *An. melas* WGS data to the AgamP4 reference genome also allowed interpretation of genetic variation in the context of chromosome location. Finally, mapping of *An. melas* data to the *An. gambiae* reference genome has been conducted in previously published studies with amplicon [26] and WGS data [27] and is an accepted method. Mapping and coverage statistics from the resulting bam files were calculated using *samtools* [37]. Variants were called for each sample using *GATK’s* HaplotypeCaller (v 4.1.4.1) to generate a VCF for each sample [38]. A combined, genotyped VCF was created for the *An. melas* samples from the Bijagós, The Gambia and Cameroon using GATK’s GenomicsDBImport and GenotypeGVCFs function [38]. The multi-sample VCF was filtered using *bcftools* (v 1.17) and *GATK’s* VariantFiltration to include chromosomal variants with the following parameters: QD > 5.0, QUAL > 30.0, SOR < 3.0, FS < 60.0, MQ > 40.0, MQRankSum > − 12.5, ReadPosRankSum > -8.0. Reads were subsequently filtered to remove reads with DP < 5.0 or GQ < 20.0, and variants were filtered to remove those with > 20.0% missing genotypes or MAF < 0.01. The final filtered VCF contained 6,767,012 variants.

### Population genetic analysis

A distance matrix was generated using PLINK (v 1.90b6.21), and principal component analyses were computed in R (v 4.3.1). A maximum likelihood tree was made using RAxML-NG (v 1.2.0) with the mitochondrial FASTA sequences for the *An. melas* samples and 43 *An. gambiae* samples from the Bijagós Archipelago [26]. The resulting maximum likelihood tree was visualised using iTOL [39]. Admixture analysis was computed using ADMIXTURE (v 1.3.0) [40]. The estimated number of ancestral populations (optimum K-value) was computed through cross-validation of 1–10 dimensions of eigenvalue decay (k = 4).

Nucleotide diversity (π) was computed for the *N* = 30 Bijagós *An. melas* mosquitoes in 20,000-bp windows on chromosome 3 L using phased filtered variants and the scikit-allel function *allel.windowed_diversity*. Tajima’s D was calculated using the function *allel.windowed_tajima_d*. Analysis was conducted with chromosome 3 L only because of the presence of large chromosomal inversions in the other chromosomes in the *An. gambiae* s.l. complex [41].

Genetic divergence between *An. melas* from the Bijagós and Cameroon was investigated using the fixation index, F_ST_. The *windowed_weir_cockerham_fst* function in scikit-allel was used to compute F_ST_ in 1-kbp windows over each chromosome (https://scikit-allel.readthedocs.io/en/stable/). Signatures of selection were investigated using three different complementary statistics: H12 [42], iHS and XP-EHH [43, 44]. Garud’s H_12_ was computed using the *moving_garud_h* function in scikit-allel, using phased biallelic SNPs in windows of 1000 SNPs. Two hundred iterations of H_12_ were calculated, and the mean value for each window was plotted. iHS was computed using phased biallelic SNPs using the *allel.ihs* function in scikit-allel (https://scikit-allel.readthedocs.io/en/stable/). Raw iHS scores were standardized using the *allel.standardize_by_allele_count* function, and *p*-values were computed and plotted. XP-EHH was calculated using phased biallelic SNPs using the *allel.xpehh* function in scikit-allel (https://scikit-allel.readthedocs.io/en/stable/). XP-EHH scores were standardised using the *allel.standardize_by_allele_count* function and plotted.

### Identification of SNPs associated with insecticide resistance

The filtered VCF was analysed to identify SNPs in four genes previously associated with resistance: *vgsc, rdl, gste2* and *ace1*. Identified variants were annotated using SnpEff (v 5.1d). The *vgsc* G2042C non-synonymous (NS) SNP was identified in 100% of allele calls in *An. melas* from all locations. This mutation has not been reported in this study as a novel NS SNP with possible association with resistance, as the identified presence in 100% of *An. melas* may have resulted from alignment to the AgamP4 genome, reflecting a species-specific mutation between our *An. melas* samples and the reference *An. gambiae*.

### Identification of structural variants

DELLY software was used to identify large structural variants (SVs) [45]. Individual bcf files were created for each sample from their bam files using DELLY [45], which were then merged and filtered to remove samples with average genome read depth of < 20 × and SVs with > 20% missingness. Filtered SVs were retained for analysis.

## Results

### Whole-genome sequence data and genetic diversity

WGS data from 30 *An. melas* from the Bijagós Archipelago were combined with an additional seven *An. melas* mosquitoes with publicly available WGS data, which were downloaded and incorporated for analysis. This included *An. melas* from Cameroon (*N* = 6) and The Gambia (*N* = 1). The combined dataset (*N* = 37) contained 6,767,012 high-quality SNPs after filtering. Average sequencing depth across the core genome ranged from 23.1 to 55.61-fold coverage, with an average of 32.61-fold coverage across all samples. The mitochondrial genome was sequenced to very high depth, averaging 2600-fold coverage and ranging from a minimum of 420- up to 5930-fold coverage.

Nucleotide diversity (π) and Tajima’s D were computed in 20,000-bp windows across chromosome 3L. This resulted in mean π = 0.003 (SD = 0.001), indicating that for any pair of mosquitoes, 0.3% of nucleotides would differ, implying low nucleotide diversity in the population. Mean Tajima’s D for chromosome 3L was calculated as D = − 1.531 (SD = 0.710), indicating an excess of rare alleles. Tajima’s D was much higher at the start of the chromosome, with mean D = 1.518 (SD = 1.334) for the first 50 windows (1Mbp) of the chromosome, suggesting balancing selection in this region (Supplementary Data 1).

### Population genetic and ancestry analysis

**Fig. 2:**
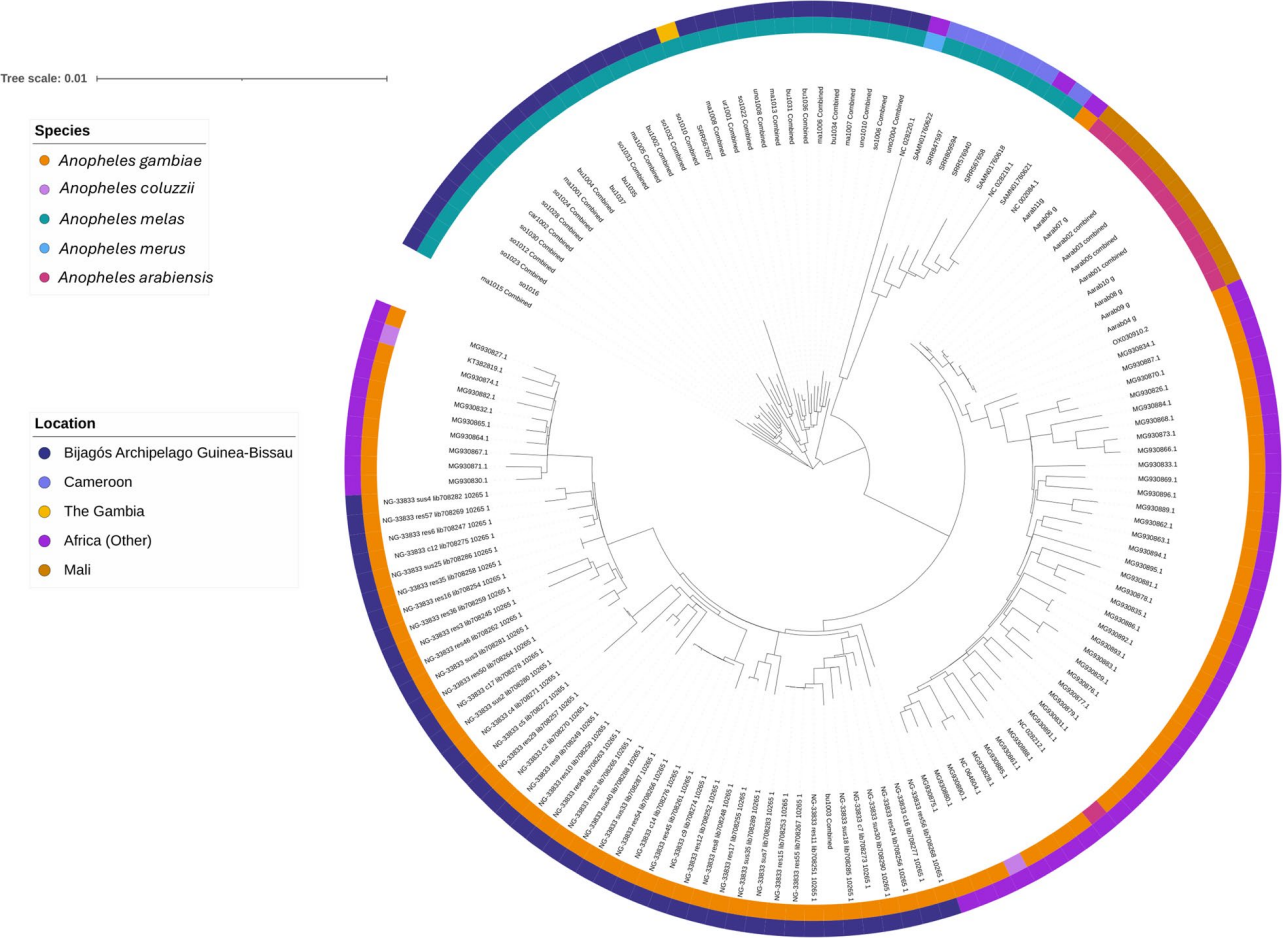
Maximum likelihood tree of whole mitochondrial sequences: *Anopheles melas* and *An. gambiae* from the Bijagós Archipelago with other species from the *An. gambiae* sensu lato species complex

A maximum likelihood tree was generated using mitochondrial sequences from the Bijagós, combined with publicly available mitochondrial sequences from additional *Anopheles* mosquitoes from across Africa in the *Anopheles gambiae* s.l. complex: *An. gambiae* s.s., *An. melas, An. merus, An. arabiensis* and *An. coluzzii* (Fig. 2). As expected, *An. melas* from the Bijagós group with the other *An. melas* specimens from The Gambia and Cameroon*.*

**Fig. 3:**
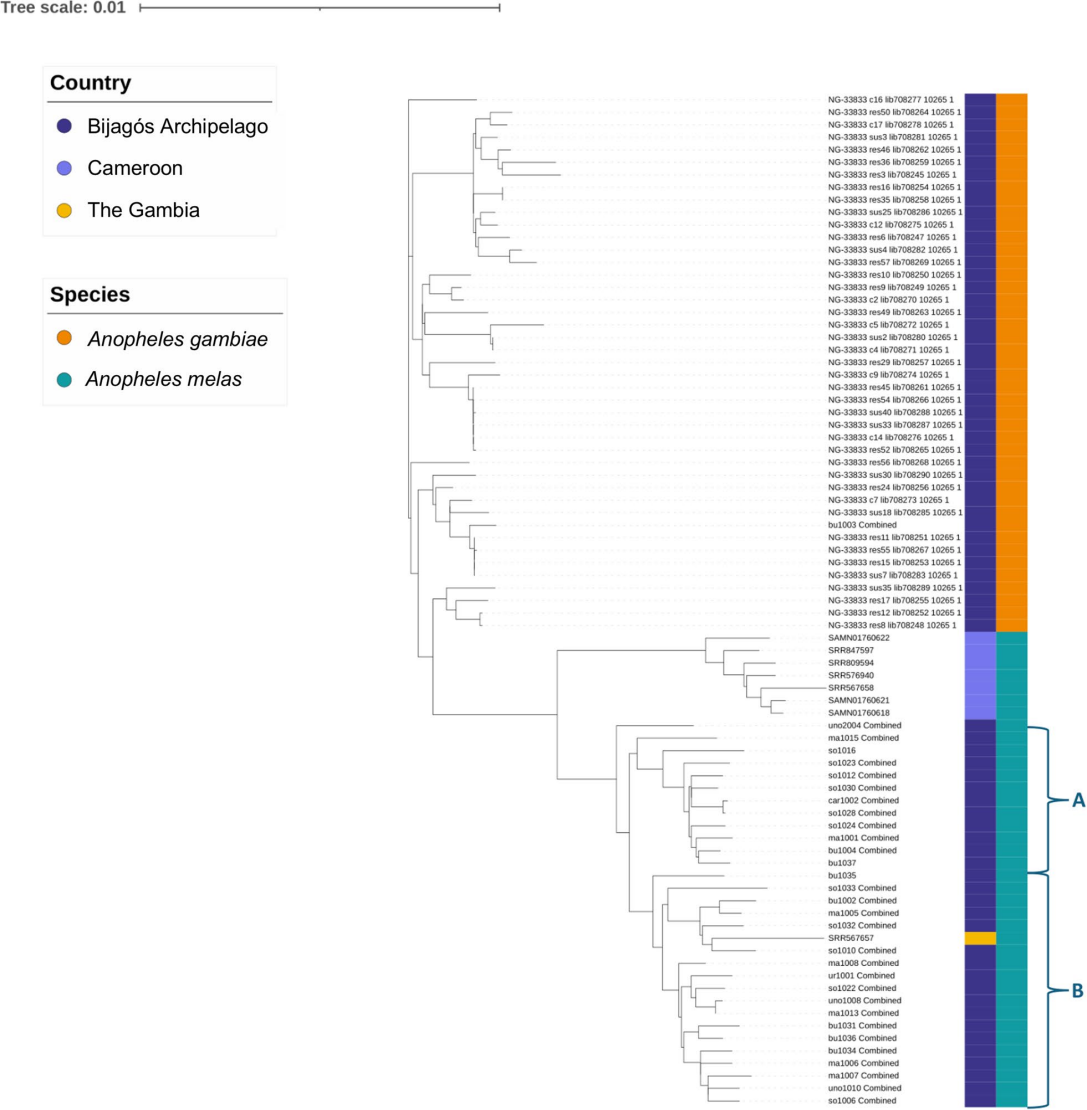
Maximum likelihood tree using *Anopheles melas* mitochondrial sequences from the Bijagós Archipelago, The Gambia and Cameroon and *An. gambiae* mitochondrial sequences from the Bijagós Archipelago. *An. melas* from the Bijagós split into two groups, labelled A and B. Support values can be seen in Supplementary Data 1

A second maximum likelihood tree was computed using whole mitochondrial sequences of *An. melas* (*N* = 37) and previously published *An. gambiae* samples from the Bijagós (*N* = 43) ([26], in review) (Fig. 3). The *An. melas* samples from the Bijagós group with the *An. melas* individual from The Gambia, and are situated next to the *An. melas* samples from Cameroon. No clustering of *An. melas* from different islands on the Bijagós was identified in the maximum likelihood tree. However, the Bijagós *An. melas* appear to form two distinct clusters, labelled A and B (Fig. 3). These clusters do not correspond to different islands of the Bijagós Archipelago, with mosquito specimens from several islands appearing in each cluster.

**Fig. 4:**
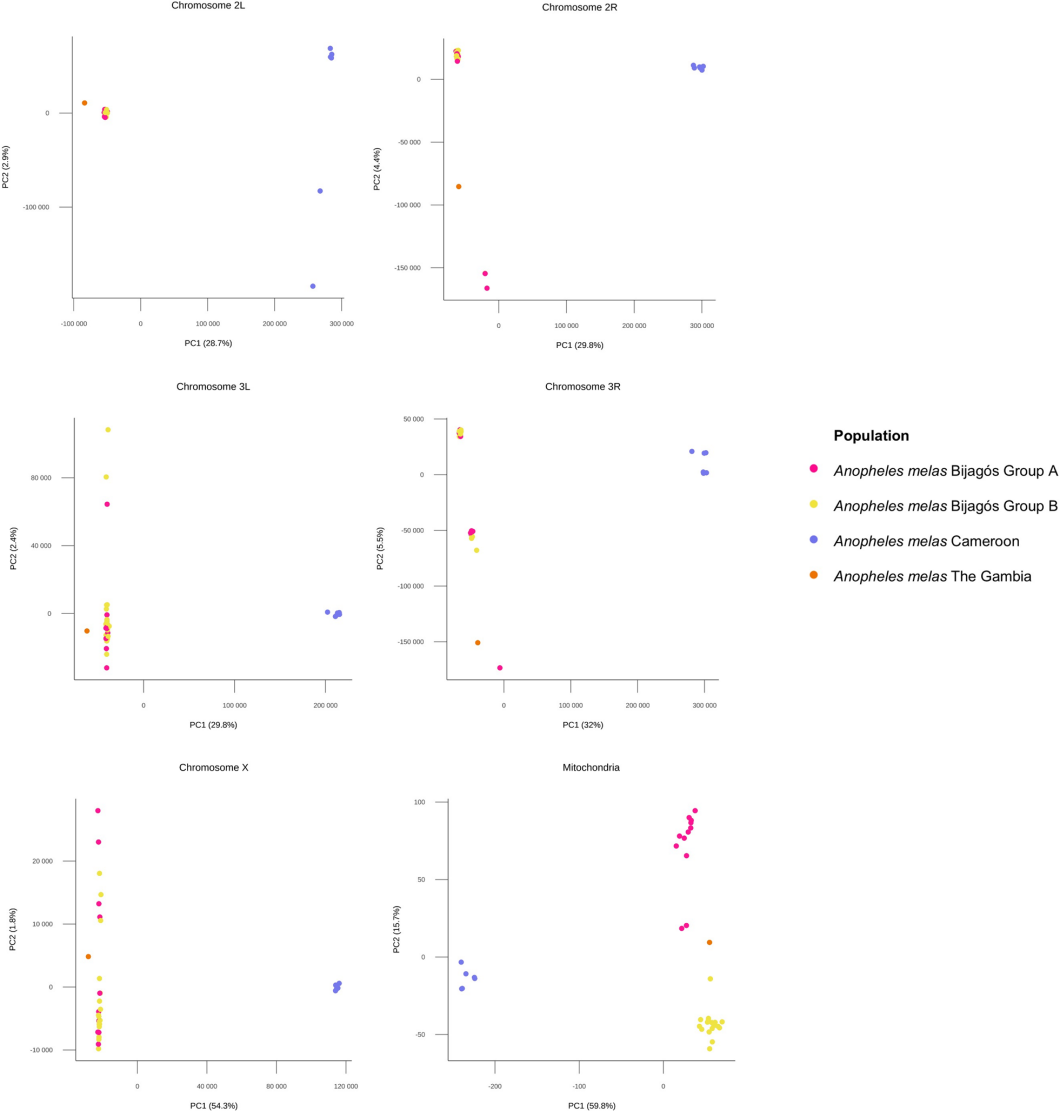
Principal component analysis for* An. melas* from the Bijagós Archipelago, Guinea-Bissau (*N* = 30), Cameroon (*N* = 6) and The Gambia (*N* = 1)

Principal component analysis (PCA) was conducted per chromosome to further investigate the relationship between the Bijagós *An. melas* mosquitoes (Fig. 4). For all chromosomes, PCA indicated that *An. melas* from the Bijagós is genetically distinct from *An. melas* samples from Cameroon and The Gambia. Comparisons with the Gambia should be treated with caution as this analysis includes only one sample. This geographic separation depicted by PC1 explains a large amount of variation for all chromosomes, ranging from 28.7% in chromosome 2 L to 59.8% in the mitochondrial genome. The clustering of *An. melas* from the Bijagós into two phylogenetic groups (A and B in Fig. 3) is supported by the PCA analysis for the mitochondrial genome. However, PCA analysis of the chromosomes 2 L, 2R and 3 L shows *An. melas* from the Bijagós clustering into one group, and chromosome X and 3R indicate some divergence but not clearly between groups A and B. Furthermore, little variance was explained by PC2 for the X and 3R chromosomes compared to the mitochondrial genome, 1.8% for chromosome X and 5.5% for chromosome 3R, compared with 15.7% for the mitochondrial genome (Fig. 4).

The clustering of Bijagós *An. melas* into groups A and B was further investigated using fixation index (F_ST_) analysis across 100-bp and 1000-bp windows. The highest F_ST_ values were on the mitochondrial genome, with a mean F_ST_ value of 0.476, compared to a mean F_ST_ value between 0.038 and 0.040 on the other chromosomes (Table 1). This indicates that most of the population differentiation is due to genetic differentiation between *An. melas* groups A and B in regions on the mitochondrial genome.

**Table 1:** F_ST_ calculated between the two clusters of *Anopheles melas*, A and B, from the Bijagós Archipelago, calculated for each chromosome and the mitochondrial genome

Chromosome	Mean (median) F_ST_ for each chromosome (100-bp windows)	Mean (median) F_ST_ for each chromosome (1000-bp windows)
2L	0.038 (0.023)	0.021 (0.014)
2R	0.039 (0.024)	0.025 (0.016)
3L	0.038 (0.024)	0.023 (0.014)
3R	0.040 (0.025)	0.025 (0.016)
X	0.038 (0.022)	0.026 (0.016)
Mt	0.476 (0.528)	0.331 (0.441)

Genes underlying peaks in F_ST_ were identified. There were seven genomic windows with an F_ST_ ≥ 0.5. These windows of the genome include the protein-coding genes detailed in Table 2. This includes genes encoding the cytochrome C oxidase subunits COX I and COX II and the NADH dehydrogenase subunits 1, 4, 4L and 5.

**Table 2:** Protein coding genes in regions of high Fst (Fst ≥ 0.5) in the mitochondrial genome, comparing the two clusters of *Anopheles melas* from the Bijagós Archipelago

Window position in mitochondrial genome (100-bp windows)	Highest F_ST_ value	Gene ID	Description
1567–1666	0.799	AGAP028364	Cytochrome C oxidase subunit (*cox1*)
1667–1766	0.528		
3467–3566	0.630	AGAP028366	Cytochrome C oxidase subunit II (*cox2*)
7067–7166	0.758	AGAP028380	NADH dehydrogenase subunit 5 (*nadh5*)
8667–8766	0.548	AGAP028382	NADH dehydrogenase subunit 4 (*nadh4*)
9667–9766	0.678	AGAP028383	NADH dehydrogenase subunit 4L (*nadh4l*)
12,367–12,466	0.748	AGAP028389	NADH dehydrogenase subunit 1 (*nadh1*)

**Fig. 5:**
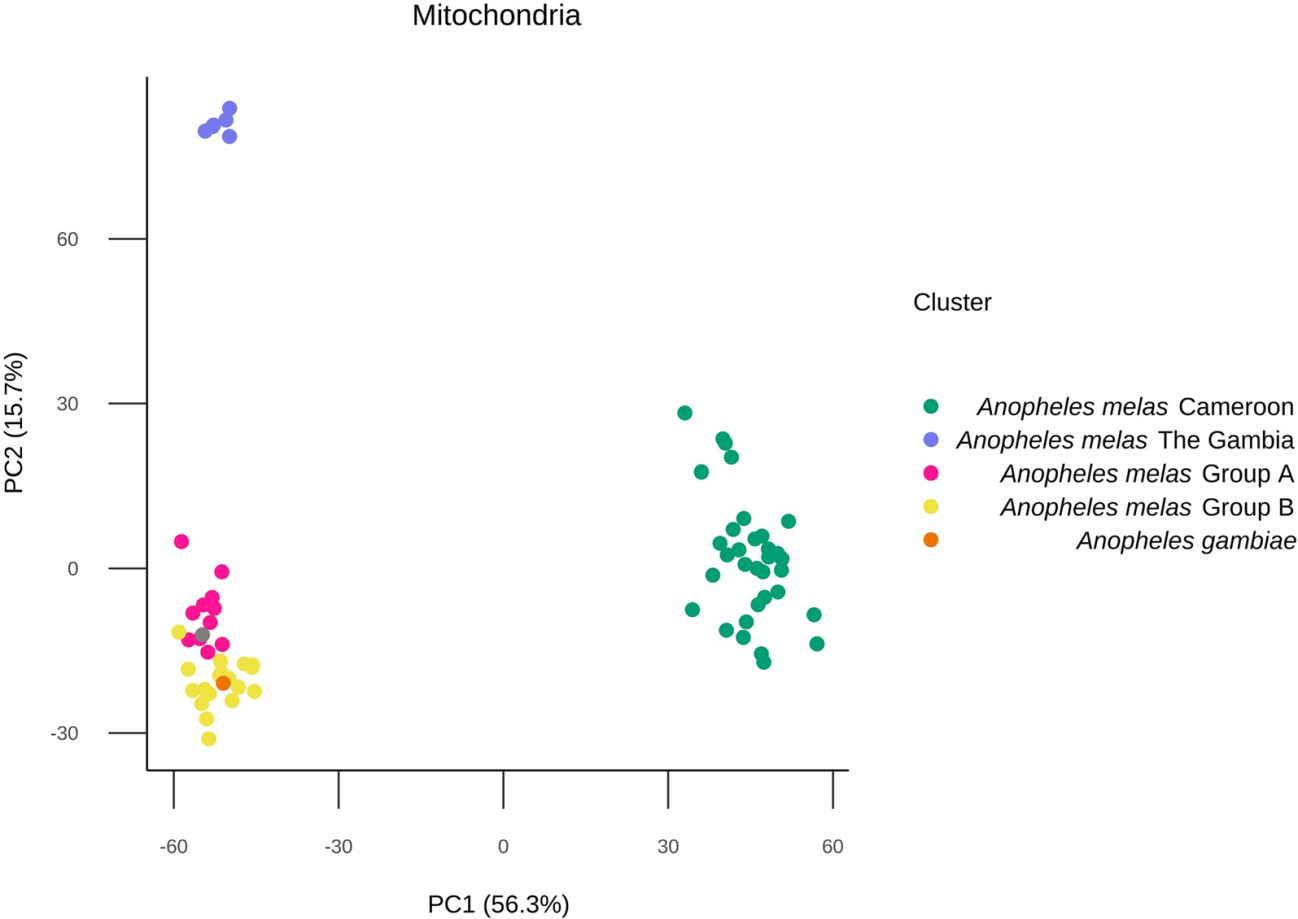
Principal components analysis comparing *Anopheles melas* and *An. gambiae* s.s. from the Bijagós Archipelago. Includes additional *An. melas* samples from Cameroon and The Gambia

Additional PCA was conducted to investigate the relationship between the genomes of the *An. melas* specimens from the Bijagós, Cameroon and The Gambia and *An. gambiae* s.s. mosquitoes from the Bijagós to investigate the possibility of hybridisation between *An. melas* and *An. gambiae* s.s. (Fig. 5). This mitochondrial PCA indicates that *An. melas* from the Bijagós separates from *Anopheles gambiae* s.s. from the Bijagós, and this relationship is also reflected in the PCA analyses of all other individual chromosomes (Supplementary Data 1). This gives no indication of hybridization between the *An. melas* and *An. gambiae* s.s. from the Archipelago.

**Fig. 6:**
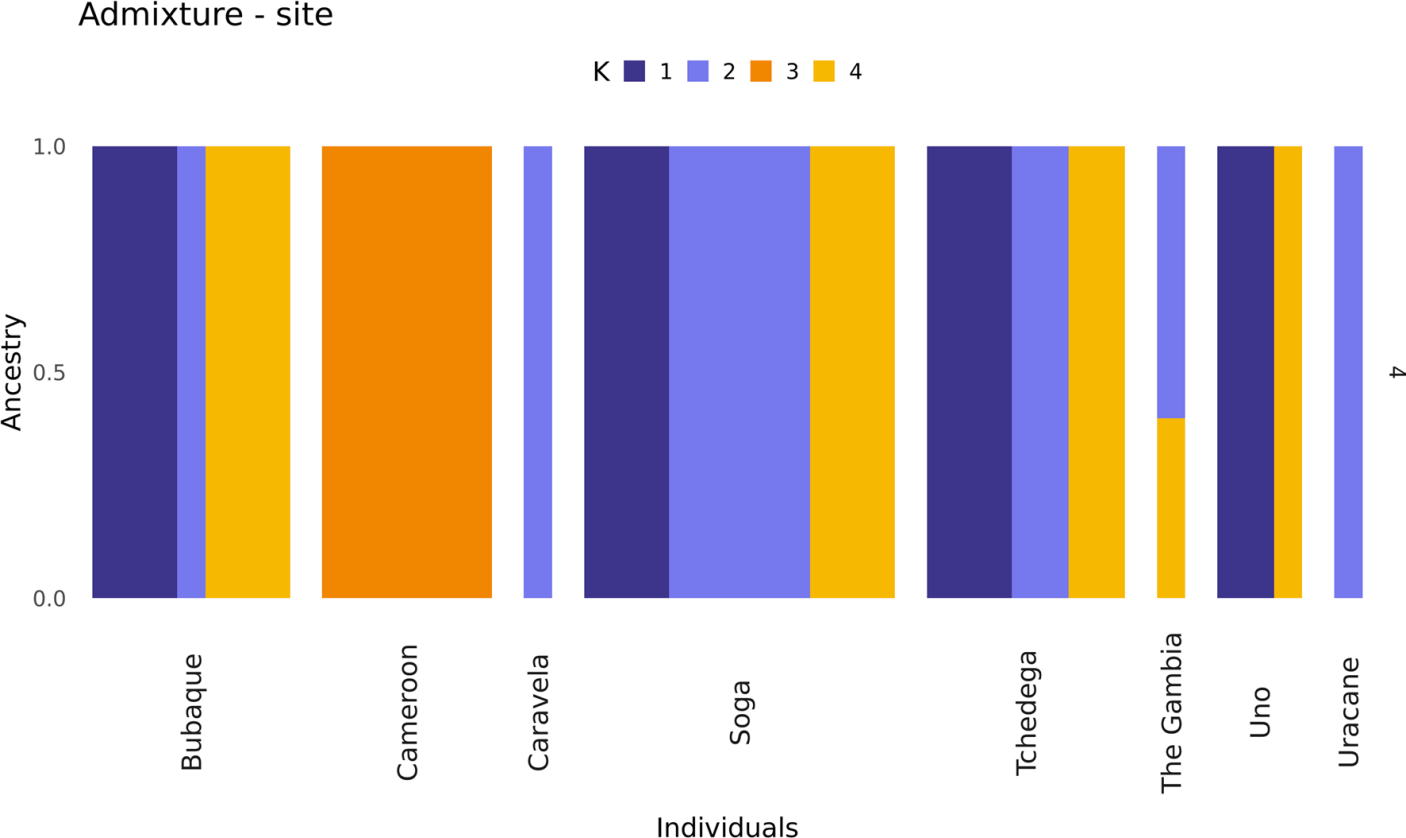
Admixture based on geographic location, K = 4. *N* = 1 The Gambia, *N* = 6 Cameroon, *N* = 30 Bijagós [*N* = 11 from Soga, 7 from Bubaque, 7 from Tchedega (Maio), 3 from Uno, 1 from Caravela and 1 from Uracane]

Admixture analysis was conducted with WGS data from the combined sample set of *N* = 37 *An. melas* available globally (Fig. 6). This admixture analysis indicated an optimum of K = 4 ancestral groups. *Anopheles melas* from the Bijagós and The Gambia had a mixture of three different K ancestries (K = 1, 2 and 4), whilst *An. melas* from Cameroon had a clearly separate ancestry (K = 3).

Population differentiation between *An. melas* from the Bijagós (*N* = 30) and *An. melas* from Cameroon (*N* = 6) was investigated using the fixation index (F_ST_) statistic (Table 3).

**Table 3:** Mean (median) F_ST_ for each chromosome, comparing *Anopheles melas* from the Bijagós and Cameroon

Chromosome	Mean (median) F_ST_ for each chromosome (1-kbp windows)
2L	0.257 (0.213)
2R	0.261 (0.216)
3L	0.270 (0.228)
3R	0.273 (0.233)
X	0.314 (0.245)
Mt	0.357 (0.305)

Fifty-nine protein coding genes were identified as overlapping windows with F_ST_ ≥ 0.9 on chromosome 2L, 72 on chromosome 2R, 51 on chromosome 3L, 56 on chromosome 3R and 56 on chromosome X (Supplementary Data 1). No genomic windows with F_ST_ ≥ 0.9 were identified on the mitochondrial genome. Consequently, windows with F_ST_ ≥ 0.6 were investigated, and 15 protein coding genes were identified (Supplementary Data 1). This included cytochrome C oxidase subunits II (*cox2*) and III (*cox3*) and NADH dehydrogenase subunits ND2 (*nadh2)*, ND3 (*nadh3*), ND4 (*nadh4*), ND4L (*nadh4l*) and ND5 (*nadh5*).

### Selection analysis identified signals of selection within *An. melas* populations

**Fig. 7:**
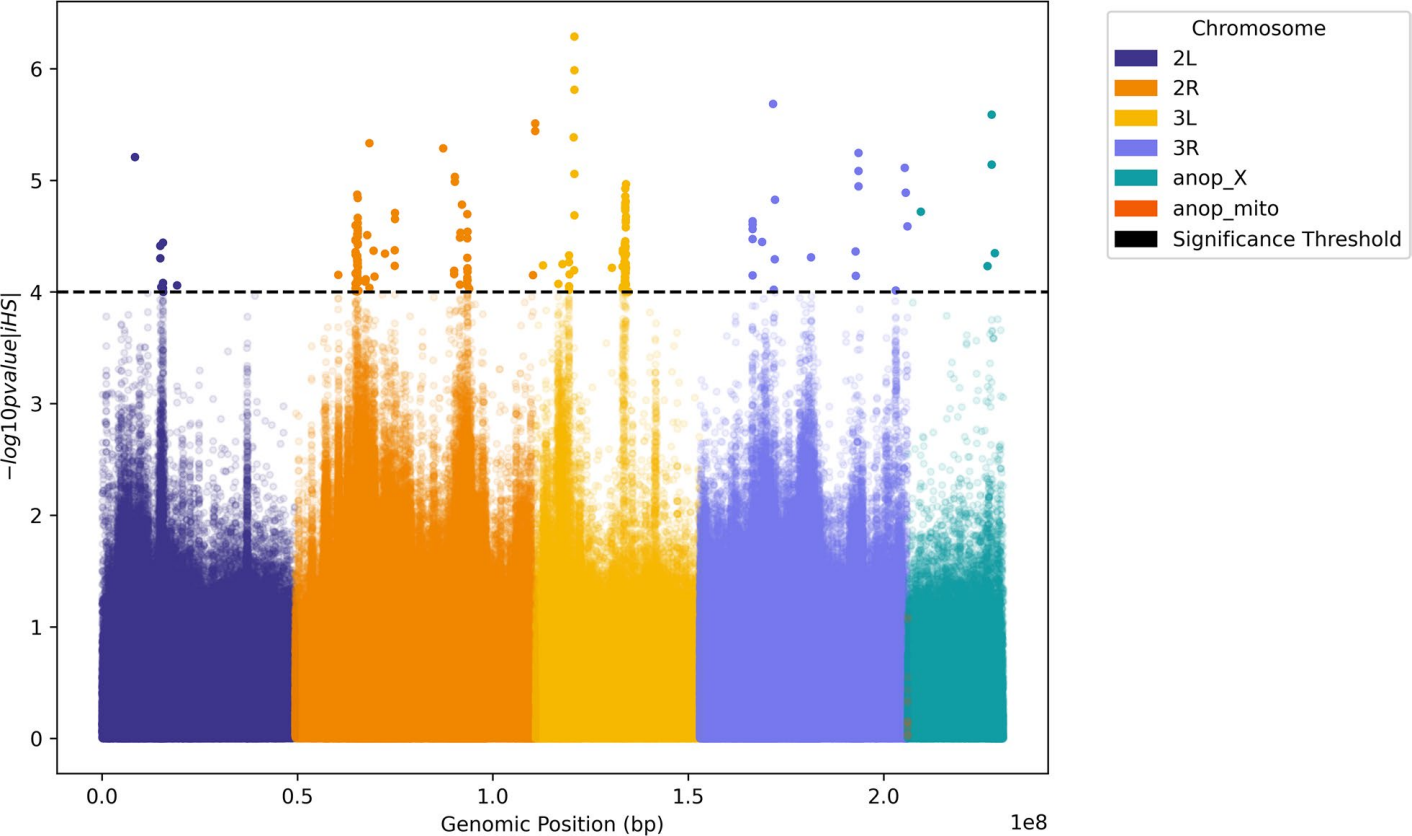
Within population selection analysis using iHS scores for *Anopheles melas* from the Bijagós Archipelago

Genome-wide selection scans of filtered variants were performed to identify signals of directional selection within and between populations of *An. melas*. Three different selection metrics were calculated: integrated haplotype score (iHS) was used to identify regions of the genome under selection within the *An. melas* Bijagós population. The cross-population haplotype homozygosity metric (XP-EHH) was used to identify regions of the genome under selection when comparing the Bijagós and Cameroon *An. melas* populations. Finally, Garud’s H12 was computed as an additional method to identify both hard and soft selective sweeps within the Bijagós *An. melas* population. In a single population analysis of *An. melas* from the Bijagós using the iHS metric [44], 194 loci were identified as having significant iHS scores (iHS ≥ 4) (Fig. 7). This included 102 SNPs in 29 different protein coding genes: 3 in chromosome 2L, 13 in chromosome 2R, 7 in chromosome 3L, 4 in chromosome 3R and 2 in chromosome X (Table 4). None of the protein coding genes identified with significant iHS scores have previously been implicated in insecticide resistance.

**Table 4:** Significant iHS scores (iHS ≥ 4) in protein coding genes in *Anopheles melas* from the Bijagós Archipelago

Chromosome	iHS score	Protein coding gene
2L	4.36	AGAP005394—MFS domain-containing protein
	4.19	AGAP005449—E3 ubiquitin-protein ligase CBL
	4.00	AGAP005450—protein coding gene—unspecified product
2R	4.15	AGAP001824—protein coding gene—unspecified product
	4.50	AGAP002118—zinc finger protein 622
	4.14	AGAP002119—dual-specificity tyrosine-(Y)-phosphorylation regulated kinase
	4.54	AGAP002123—axin
	4.09	AGAP002235—GATA-binding protein 4/5/6
	4.11	AGAP002243—ankyrin repeat and FYVE domain-containing protein 1
	4.51	AGAP002274—protein coding gene—unspecified product
	4.69	AGAP002299—XK-related protein
	4.37	AGAP002336—Ig-like domain-containing protein
	4.34	AGAP002573—GTPase-activating Rap/Ran-GAP domain-like protein 3
	4.49	AGAP002677—coiled-coil domain-containing protein lobo homolog
	4.78	AGAP029573—protein coding gene—unspecified product
	4.15	AGAP004670—protein coding gene—unspecified product
3L	4.24	AGAP010344—solute carrier family 26
	4.07	AGAP010536—nucleolar complex protein 2
	5.39	AGAP029471—protein coding gene—unspecified product
	6.03	AGAP029721—fibrinogen C-terminal domain-containing protein
	4.22	AGAP011223—fibrinogen C-terminal domain-containing protein
	4.30	AGAP011379—Frizzled receptor
	4.46	AGAP011384—protein coding gene—unspecified product
3R	4.45	AGAP008712—solute carrier organic anion transporter family member
	4.02	AGAP008826—protein coding gene—unspecified product
	4.15	AGAP009716—cadherin
	5.11	AGAP010295—Ca_chan_IQ domain-containing protein
X	4.23	AGAP001046—Abl interactor 2
	4.35	AGAP001064—transmembrane emp24 domain-containing protein 10 precursor

**Fig. 8:**
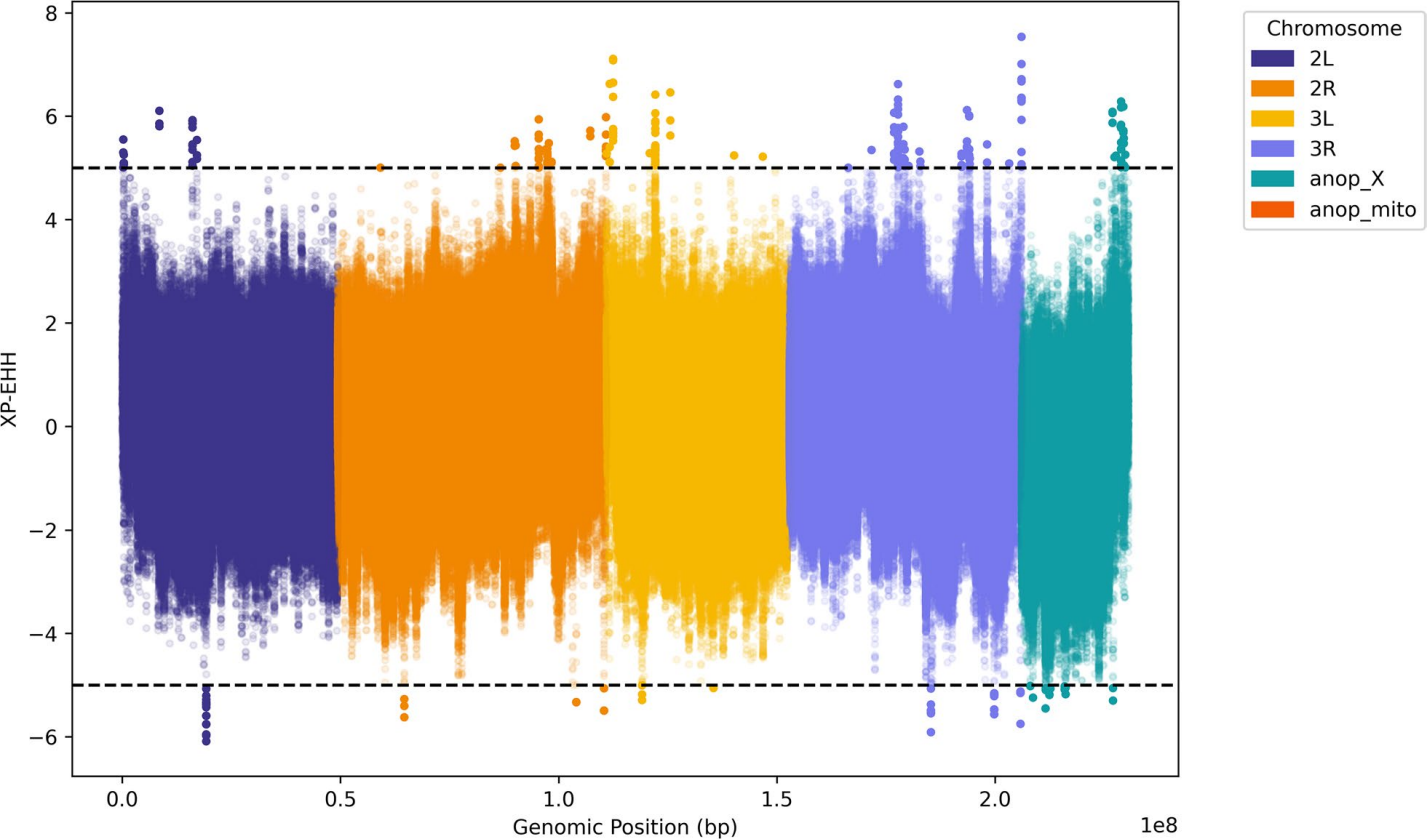
Cross population selection analysis using XP-EHH metric between *Anopheles melas* from the Bijagós and Cameroon

Cross-population analysis using XP-EHH was conducted between *An. melas* from the Bijagós and Cameroon (Fig. 8). More positive scores indicate positive selection in the Bijagós *An. melas* population (significant at XP-EHH ≥ 5), whereas more negative scores indicate positive selection in the Cameroon *An. melas* population (significant at XP-EHH ≤ − 5). Protein coding genes containing SNPs under positive selection in the Bijagós population (Table 5) and the Cameroon population (Table 6) are detailed.

**Table 5:** Protein coding genes containing SNPs under positive selection in the Bijagós *Anopheles melas* population, relative to the Cameroon *An. melas* population, with XP-EHH scores ≥ 5

Chromosome	XP-EHH score	Protein coding gene
2L	5.54	AGAP005483—transcription factor grauzone
	5.32	AGAP005562—RING-box protein 2
2R	5.00	AGAP001773—putative allatostatin receptor 2
	5.36	AGAP003997—casein kinase 1, gamma
	5.20	AGAP004032—alpha-mannosidase
3L	5.28	AGAP010310—eukaryotic peptide chain release factor subunit
	5.56	AGAP010324—Groucho
	5.29	AGAP029721—fibrinogen C-terminal domain-containing protein
	5.45	AGAP010814—TEP6 thioester-containing protein 6
3R	5.52	AGAP028085—LITAF domain-containing protein
	5.39	AGAP009115—phosphatidylinositol phospholipase C, beta
	5.08	AGAP009253—protein coding gene—unspecified product
	5.17	AGAP009723—cadherin
	5.28	AGAP029826—Tox-SGS domain-containing protein
X	5.41	AGAP001073—protein coding gene—unspecified product

**Table 6:** Protein coding genes containing SNPs under positive selection in the Cameroon *Anopheles melas* population, relative to the Bijagós *An. melas* population, with XP-EHH scores ≤ − 5

Chromosome	XP-EHH score	Protein coding gene
2R	− 5.35	AGAP013143—Rho GTPase-activating protein 26
3L	− 5.15	AGAP010663—female reproductive tract protease GLEANR_2575
3R	− 5.48	AGAP009415—lysophosphatidate acyltransferase
	− 5.30	AGAP009970—Cullin-associated NEDD8-dissociated protein 1
X	− 5.02	AGAP000116—Rab11 family-interacting protein 1/2/5
	− 5.24	AGAP013158—protein coding gene—unspecified product
	− 5.45	AGAP000304—protein twisted gastrulation
	− 5.07	AGAP000311—UNC93-like protein MFSD11
	− 5.19	AGAP000356—SCP domain-containing protein
	− 5.06	AGAP000547—rabenosyn-5
	− 5.11	AGAP000567—protein coding gene—unspecified product

Garud’s H_12_ was used to identify signatures of recent positive selection in the Bijagós *An. melas* population [42]. H_12_ was computed per chromosome, and no clear peaks of selection were identified on any chromosome (Supplementary Data 1).

### Detection of structural variants

Structural variants (SVs) were identified in the Bijagós *An. melas* mosquito population using DELLY software [45] and were discovered in relation to the AgamP4 (*An. gambiae)* reference genome. A total of 113,121 SVs were identified across the whole genome following quality control filtering. Of these 113,121 filtered SVs, 116 were identified in genes associated with insecticide resistance or in gene families associated with insecticide metabolism. This included 48 deletions, 38 inversions, 22 insertions and 8 duplications.

SVs were annotated using SnpEff [46]. Of the 48 identified deletions, four were annotated as having high impact. This included one deletion (40 bp) in chromosome 2R, which was found at 3% allelic frequency and resulted in a frameshift in *cyp6p15p* and the up- or downstream modification of *cyp6aa2, coeae60, cyp6p3* and *cyp6p5*. Two high-impact deletions on chromosome 2 L (766,776 bp) and 3R (714,267 bp) resulted in feature ablation of multiple genes and were found in all *An. melas* samples from the Bijagós and Cameroon, indicating that these deletions may be species specific to *An. melas* and could have been identified as a result of aligning to the AgamP4 reference genome. The fourth high-impact deletion was identified in chromosome X (505,172 bp) at 47.9% allelic frequency in the Bijagós population and 28.6% in the Cameroon population, which resulted in the deletion of multiple genes including AGAP000817, AGAP000816 and AGAP013474 (protein coding genes with unspecified products). Two deletions were annotated as having moderate impact. The first was found at 1.7% allelic frequency in the *cyp6m4* gene on chromosome 3R (575 bp). The second was on chromosome 2R (38 bp) in AGAP013202 and was identified in all *An. melas* samples from the Bijagós and Cameroon so may be *An. melas* specific.

Of the 22 insertions identified, 5 were in introns, 8 were in intergenic regions, 4 were downstream gene variants, and 5 were upstream gene variants. None of these insertions were identified as having high impact, and 14 were identified in all of the Bijagós *An. melas* isolates, indicating that they may be species specific insertions.

Of the eight identified duplications, five were annotated as having high impact. Two of these duplications were identified on chromosome 2R at 2% allelic frequency. The first leads to bidirectional gene fusion between *cyp6p4* and the solute carrier family 8 sodium/calcium exchanger (AGAP002859), and the second leads to bidirectional gene fusion between AGAP002876 (encoding a DNA glycosylase) and AGAP002877 (encoding a tetratricopeptide repeat protein). Two other high-impact duplications were identified on chromosome 3R. The first was found at 2% allelic frequency and leads to gene fusion between *cyp6m2* and *cyp6m3*, whereas the second occurred at 4% allelic frequency and results in a premature stop codon in *gste1*. Finally, a large duplication on chromosome X between genomic positions 14,939,828 and 15,316,733 (376,905 bp) was identified to have high impact. All Bijagós *An. melas* and six of the seven Cameroon *An. melas* were heterozygous for this duplication, which spans multiple protein coding genes including the cytochrome P450 *cyp9k1*. In addition, one moderate impact duplication was identified at 1.7% allelic frequency in *cyp9k1* on chromosome X.

Of the 38 identified inversions, 8 were annotated as having high impact. This included three different inversions in the *vgsc* gene on chromosome 2L found at 10%, 4% and 2% allelic frequency, respectively, two different inversions in the AGAP009189 gene on chromosome 3R found at 2% allelic frequency, one inversion in *gste8* on chromosome 3R found at 4% allelic frequency and one inversion in the zf-C3Hc3H domain-containing protein (AGAP029190) gene in chromosome 2R found at 6% frequency. One large, high-impact, inversion in chromosome 2L spanned from genomic positions 2,161,433 to 2,666,161 and led to bidirectional gene fusion between AGAP004717 and AGAP029667 (protein coding genes with unspecified products). All *An. melas* Bijagós and Cameroon mosquitoes were homozygous alternate for this inversion, indicating that this could be a species-specific inversion in *An. melas* identified through alignment to the AgamP4 reference genome. One additional moderate impact inversion was identified on chromosome 3L at 15% allelic frequency, impacting the DNA-directed RNA polymerase III subunit RPC1 (AGAP004703), compass component SPP1 (AGAP004704), arginyl-tRNA synthetase (AGAP004708) and five protein coding genes with unspecified products.

### Identification of non-synonymous SNPs in resistance genes

We investigated the presence of target site mutations in the *vgsc, gste2, rdl* and *ace1* genes, which have previously been associated with insecticide resistance (Supplementary Data 1). In total, we identified 28 non-synonymous mutations in these resistance genes (Table 7). Of these, the only mutation that has been reported previously is *vgsc* M490I, which we identified at an allelic frequency of 2% and is highlighted with a † symbol in Table 7. This mutation was previously identified in *An. gambiae* samples from Kenya, where it was found to potentially be under selection [47]. To our knowledge, none of the other missense mutations identified here have previously been reported in the *An. gambiae* s.l. complex. The *vgsc* G2046S mutation was fixed in the Bijagós *An. melas* population (100% allelic frequency) and was identified in one *An. melas* sample from The Gambia (100% allelic frequency) but was not found in any *An. melas* samples from Cameroon (0% allelic frequency).

**Table 7:** Non-synonymous SNPs identified in the Bijagós *Anopheles melas* mosquito population in resistance genes

Gene	Chromosome	Position^a^	Average read depth	SNP^b^	Transcript	Homozygous reference	Heterozygous	Homozygous alternate	Allelic frequency
*vgsc*	2L	2,358,197	31.37	S14C	AGAP004707-RD	21	9	0	15%
		2,390,341	59.67	T309A	AGAP004707-RD	29	1	0	2%
		2,390,449	63.67	G317D	AGAP004707-RD	28	2	0	3%
		2,390,472	61.37	S325T	AGAP004707-RD	29	1	0	2%
		2,390,737	56.73	L377Q	AGAP004707-RD	29	1	0	2%
		2,391,309	69.53	E429K	AGAP004707-RD	1	29	0	48%
		2,400,071	33.63	^†^M490I	AGAP004707-RD	29	1	0	2%
		2,416,868	33.47	L754M	AGAP004707-RD	28	2	0	3%
		2,424,377	31.57	N1123D	AGAP004707-RD	28	2	0	3%
		2,429,991	33.37	E1628D	AGAP004707-RD	29	1	0	2%
		2,431,184	36.30	R1975Q	AGAP004707-RD	29	1	0	2%
		2,431,396	33.33	G2046S	AGAP004707-RD	0	0	30	100%
*gste2*	3R	28,597,858	34.53	I187F	AGAP009194-RA	29	1	0	2%
		28,597,879	34.47	H180Y	AGAP009194-RA	29	1	0	2%
		28,597,905	35.83	G171D	AGAP009194-RA	23	7	0	12%
		28,598,032	36.73	D129N	AGAP009194-RA	29	1	0	2%
		28,598,526	28.37	E19K	AGAP009194-RA	29	1	0	2%
		28,598,573	29.83	N3S	AGAP009194-RA	4	11	15	68%
*rdl*	2L	25,433,558	28.60	P474Q	AGAP006028-RA	29	1	0	2%
		25,433,561	27.70	P475Q	AGAP006028-RA	29	1	0	2%
*ace1*	2R	3,489,216	34.23	E2K	AGAP001356-RA	29	1	0	2%
		3,489,310	34.50	P33Q	AGAP001356-RA	29	1	0	2%
		3,489,391	36.37	A60V	AGAP001356-RA	29	1	0	2%
		3,493,397	28.50	P644T	AGAP001356-RA	26	3	1	8%
		3,493,401	28.77	N645S	AGAP001356-RA	28	1	0	2%
		3,493,715	28.47	A714V	AGAP001356-RA	28	1	0	2%
		3,493,759	31.03	L729F	AGAP001356-RA	29	1	0	2%
		3,493,771	31.00	V733I	AGAP001356-RA	28	2	0	3%

^a^SNP positions correspond to the AgamP4 reference genome. ^b^Codon numbering according to transcript in the AgamP4 reference genome. M490I has been highlighted with a ^†^ symbol

## Discussion

*Anopheles melas* is highly abundant on the Bijagós Archipelago of Guinea-Bissau and may have a role in malaria transmission [4] (Pretorius et al. 2024, in review). However, the population structure and insecticide resistance status of this malaria vector are not well understood. This study used WGS data from 30 *An. melas* from across the Archipelago to investigate genetic diversity, population structure and signatures of selection in insecticide resistance genes within this vector population.

Maximum likelihood trees generated using the whole mitochondrial genome showed that *An. melas* from the Bijagós split into two groups. Mosquito samples were collected from six different islands in the Archipelago, but this split was not associated with sampling island, and analyses indicated that the clustering of *An. melas* into two groups was due to genetic differentiation on the mitochondrial genome. The protein coding genes underlying the peaks in F_ST_ on the mitochondrial genome included the cytochrome C oxidase subunits *cox1* and *cox2* and the NADH dehydrogenase subunits *nadh1*, *nadh4*, *nadh4L* and *nadh5*. Both *cox1* and *nadh4* have previously been used to investigate phylogenetic relationships within multiple vector complexes and between cryptic species, including the *An. gambiae* s.l. complex [48–50]. Further investigation with a larger sample size of *An. gambiae* s.l. complex mosquitoes from the Bijagós Archipelago will help to understand the clusters observed.

The computed maximum likelihood trees indicate that *An. melas* from the Bijagós are closely related to *An. melas* from The Gambia and Cameroon. The level of genetic differentiation between *An. melas* from the Bijagós and Cameroon was higher than previously identified for other species of *An. gambiae*. Average F_ST_ across chromosomes 3L and 3R between the Bijagós and Cameroon *An. melas* was 0.27. However, F_ST_ across these chromosomes was previously identified at a tenfold lower score of 0.028 between mainland Guinea-Bissau and Cameroon *An. gambiae* [25]. Higher than expected levels of genetic differentiation have previously been identified between *An. melas* populations using whole-genome data. A previous study by Deitz et al. found that divergence between large *An. melas* clusters along the West African coast were due to high levels of differentiation across the whole genome, indicative of allopatric separation [27]. Nucleotide diversity was similar to that previously identified for *An. melas* from Bioko Island, Equatorial Guinea (π = 0.0034) [27], and was lower than the average nucleotide diversity calculated for *An. gambiae* sampled from 15 locations across Africa (π = 0.015) [25]. Ancestry analysis using a combined database of these *An. melas* samples indicated K = 4 ancestral populations, with *An. melas* samples from the Bijagós and The Gambia sharing ancestries K = 1, 2 and 4 and Cameroon *An. melas* samples sharing a distinct K = 3 ancestry. There was no clear distinction between the ancestries of *An. melas* from different islands on the Archipelago, indicating historical gene flow between the islands, despite the geographical distance between islands being greater than the distance *An. gambiae* s.l. are known to disperse [51]. This is supported by a previous study, which identified extensive gene flow between *Anopheles* s.s. on the Bijagós Archipelago and mainland Guinea-Bissau [52]. Fixation index analysis identified 59 protein coding genes with high genetic differentiation between *An. melas* from the Bijagós and Cameroon, including the *cyp307a1* gene (AGAP001309) on chromosome X, which is a member of the cytochrome P450 gene family associated with metabolic resistance to insecticides [53, 54]. This analysis was conducted with a large sample size disparity, with 30 samples from the Bijagós vs. 6 samples from Cameroon. Additional WGS data are required to further investigate this genetic differentiation.

Genome-wide selection scans were computed to identify signatures of selection across the genome. Within-population analysis of Bijagós *An. melas* using the iHS statistic identified signatures of directional selection in 29 protein coding genes, none of which have previously been associated with insecticide resistance. Cross-population analysis between *An. melas* from the Bijagós and Cameroon using the XP-EHH metric identified 15 protein coding genes within the Bijagós population undergoing positive selection compared to the Cameroon population. This included the gene encoding *tep6*, a thioester-containing protein in the same family as *tep1*, which is implicated in *An. gambiae* resistance to parasite infection [55]. This analysis also identified 11 protein coding genes undergoing positive selection in the Cameroon population compared to the Bijagós population, none of which have previously been associated with insecticide resistance. Genome-wide selection scans using the H_12_ statistic did not identify any clear selective sweeps in the Bijagós *An. melas* genome, in contrast to our previous study of *An. gambiae* s.s. collected during 2022 from Bubaque Island, where H_12_ analysis identified two distinct selective sweeps on chromosomes X and 2R spanning multiple cytochrome-P450 genes involved in insecticide metabolism ([26], in review). The absence of selective sweeps in insecticide resistance-associated genes in the *An. melas* genome suggests that this species may be under less selective pressure from insecticides than *An. gambiae* on these islands.

Structural variants (SVs) were analysed in the Bijagós *An. melas* population in relation to the *An. gambiae* AgamP4 reference genome. One deletion identified in chromosome 2R at 3% allelic frequency resulted in a frameshift in *cyp6p15p* and modification of *cyp6aa2, coeae60, cyp6p3* and *cyp6p5.* These *cyp6p* genes are associated with metabolic insecticide resistance in mosquitoes [53, 54, 56, 57]. Another variant resulted in the duplication of multiple protein coding genes including the cytochrome P450 *cyp9k1*, which metabolises deltamethrin and has been associated with pyrethroid resistance in *An. coluzzii* populations following vector control interventions [21, 22]. Furthermore, *cyp9k1* duplications have previously been identified in *An. gambiae* s.l. complex mosquitoes from mainland Guinea-Bissau ([23], Supplementary S7) [23]. Copy number variants resulting in gene duplication are under positive selection in the *An. gambiae* s.l. complex [23], and CNVs leading to increased expression of metabolic genes have been shown to increase insecticide metabolism, leading to insecticide resistance [56, 58–60]. Eight high-impact inversions were identified, including three inversions in the *vgsc* gene associated with resistance to DDT and pyrethroids [17, 18, 56, 61], and one inversion in the *gste8* gene, which is in the same gene family as *gste2*, which encodes a DDT-detoxifying enzyme [62].

Analysis of non-synonymous SNPs in insecticide resistance genes identified the *vgsc* M490I mutation in the Bijagós *An. melas* population at low frequency, which has previously been reported in *An. gambiae* in Kenya as under possible directional selection [47]. No other SNPs previously associated with insecticide resistance were found. In our previous study of amplicon data from *An. melas* from the Archipelago collected in the same year, three SNPs previously associated with insecticide resistance were identified. These were *vgsc* L995F (2.14% allelic frequency), N1570Y (1.12% allelic frequency) and A1746S (0.76% allelic frequency) [26]. However, these SNPs were identified at very low frequency and found at significantly lower frequency in *An. melas* than in *An. gambiae* s.s. Therefore, the absence of these SNPs in our dataset is not unexpected.

The absence of insecticide resistance-associated SNPs in *An. melas* further suggests that this species is under less insecticide resistance selection pressure than *An. gambiae* on the Bijagós Archipelago. This is supported by a previous study in Equatorial Guinea, where no insecticide resistance mutations were identified in *An. melas* [12]. This may be because *An. melas* is biting people outdoors, circumventing exposure to the insecticides in ITNs, or because *An. melas* may be feeding mostly on non-human hosts [6]. In an entomological survey on the Bijagós in 2019, a greater proportion of *Plasmodium*-positive *An. melas* were caught in the outdoor than indoor traps. However, *Plasmodium*-positive *An. melas* were also caught in indoor traps, indicating human-host seeking indoors and outdoors (Pretorius et al. 2024, in review). As ITNs are the only vector control method used in the Bijagós, outdoor biting would reduce selection pressure for resistance evolution in this species. Whilst maintaining susceptibility to insecticides is positive, preferential outdoor biting by *An. melas* may present further issues for vector control on the islands as conventional ITNs and IRS may not be as effective. This is supported by studies in Equatorial Guinea which identified high levels of outdoor biting by *An. melas* [63]. Alternatively, the absence of insecticide resistance-associated mutations and selective sweeps in *An. melas* found in this study may be because this species is evolving separate molecular mechanisms of resistance to *An. gambiae* s.s. Notably, *Anopheles* mosquitoes are among the most genetically diverse eukaryotic organisms known [25], and it is plausible that different species may evolve unique molecular pathways for resistance. Further investigation using additional sampling, phenotypic bioassays, synergist-insecticide bioassays and ‘omics studies should be undertaken to understand the resistance status and molecular mechanisms of resistance in this understudied malaria vector.

The main limitations of this study are the absence of phenotypic insecticide resistance data for *An. melas* and that the current *An. melas* reference genome necessitated aligning our *An. melas* WGS data to the *An. gambiae* (AgamP4) reference genome. The AgamP4 reference genome is of higher quality, and 92.8% of *An. melas* reads mapped successfully to AgamP4 compared to 79.3% mapping to the poorer quality AmelC2 reference genome, giving us confidence in our approach. However, future analyses would benefit from a chromosome-level reference genome assembly for *An. melas*, particularly as all structural variants were discovered in relation to the AgamP4 reference. Furthermore, the genes discussed in this study have been annotated from the *An. gambiae* reference genome, and though these genes are likely to have very similar functions in *An. melas*, they could play different roles.

## Conclusions

In conclusion, using WGS data, this study identifies two separate phylogenetic clusters of *An. melas* on the Bijagós Archipelago of Guinea-Bissau because of genetic differentiation on the mitochondrial genome. Structural variants encompassing genes that could be involved in metabolic insecticide resistance were identified. However, common SNPs associated with insecticide resistance in *An. gambiae* s.s. were absent in the *An. melas* population, and there were no clear signatures of selection in known insecticide-resistance genes. This suggests that *An. melas* may experience less selective pressure for insecticide resistance evolution than *An. gambiae*, potentially through biting outdoors and circumventing selection pressure from ITNs or because they are feeding primarily on non-human hosts. Further investigations using larger data sets and phenotypic bioassays are required.

## Supplementary Information

Additional file 1

## Data Availability

The raw sequence data generated and analysed during this study are available in the European Nucleotide Archive (project ID: PRJEB75927, accession numbers ERS20101387 to ERS20101416).
