# Supplementary material for: Whole genome sequence analysis of population structure and insecticide resistance markers in Anopheles melas from the Bijagós Archipelago, Guinea-Bissau
Source: Parasit Vectors. 2024 Sep 18;17:396. doi: 10.1186/s13071-024-06476-2 (PMC11412053; doi:10.1186/s13071-024-06476-2)
Supplement: Supplementary file 1 — Additional file 1 [file 13071_2024_6476_MOESM1_ESM.docx]

**Supplementary Data**


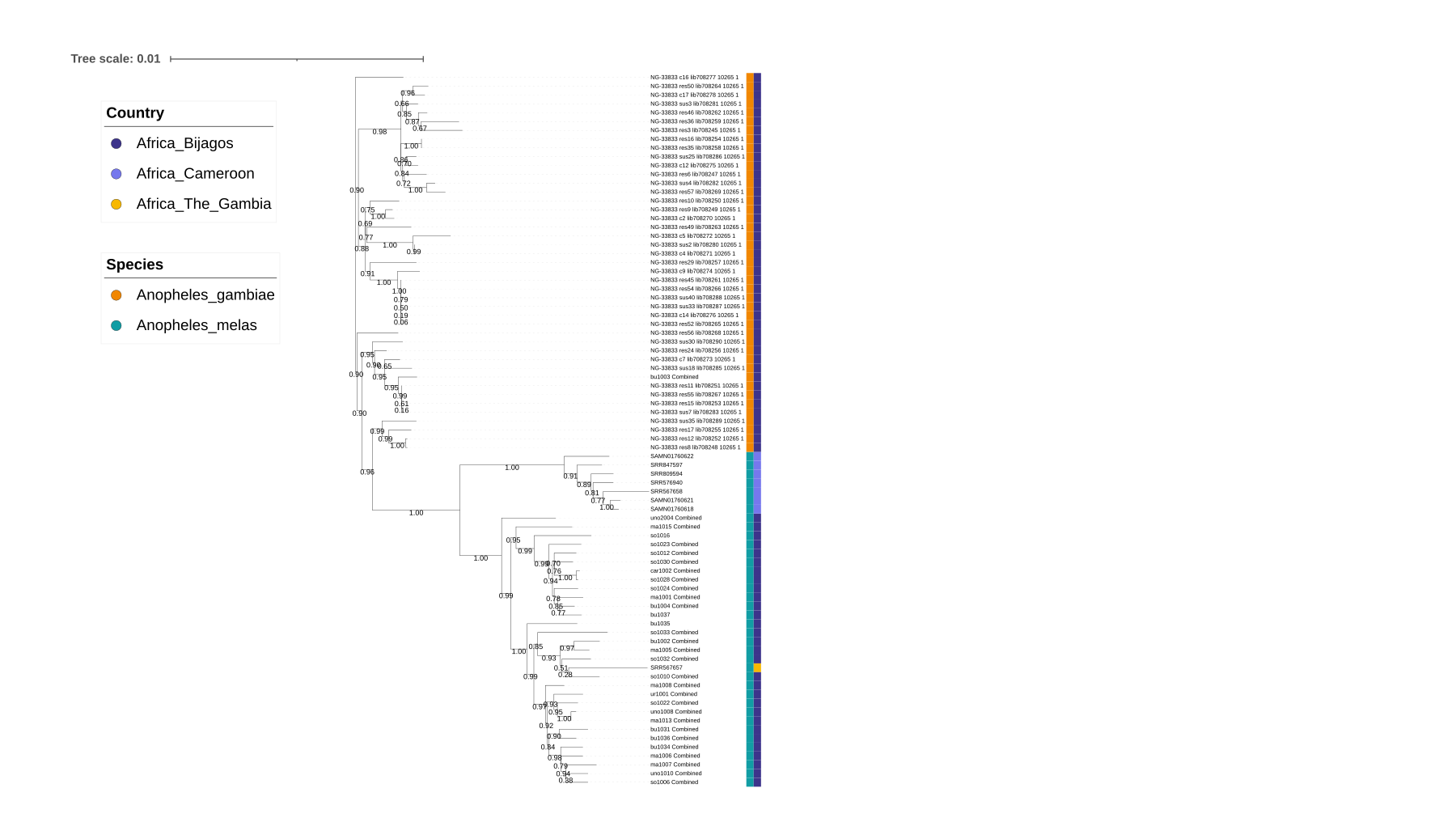


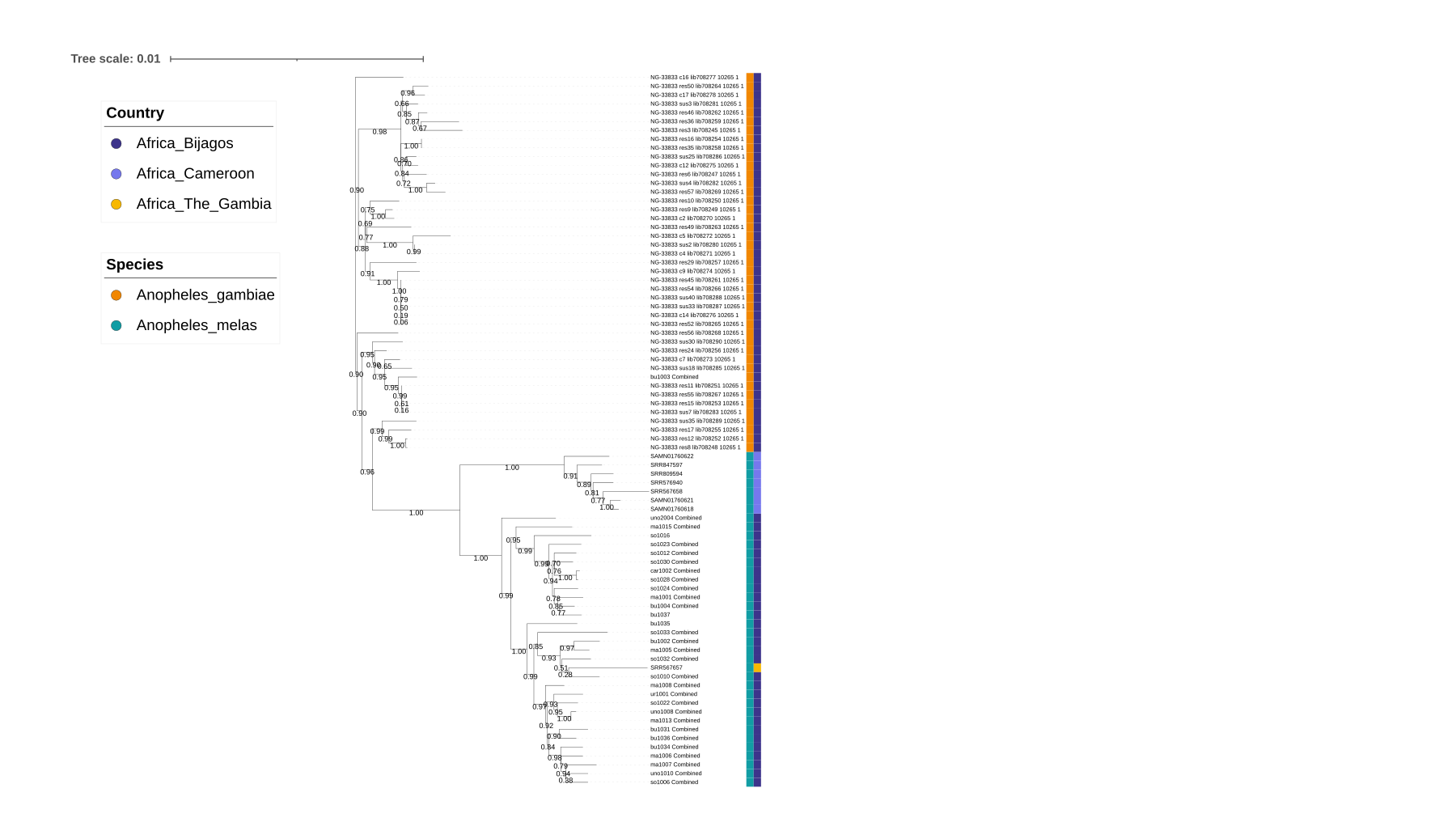


Supplementary Figure 1: Maximum likelihood tree of whole mitochondrial sequences: An. melas and An. gambiae from the Bijagós Archipelago with other species from the An. gambiae sensu lato species complex, including bootstrap support values.

Supplementary Table 1: Single Nucleotide Polymorphisms (SNPs) with F_ST_ ≥ 0.9 for chromosome 2L: comparison between An. melas from the Bijagós Archipelago with An. melas from Cameroon.

| **Chromosome** | **Window (Genomic bps)** | **F_ST_ Value** | **Gene ID** | **Protein coding gene** |
| --- | --- | --- | --- | --- |
| 2L | 2897239-2898238 | 0.905 | AGAP029113 | protein coding gene - unspecified product |
|  | 4429239-4430238 | 0.937 | AGAP004847 | Class B Scavenger Receptor (CD36 domain) |
|  | 4956239-4957238 | 0.937 | AGAP004886 | protein coding gene - unspecified product |
|  | 6891239-6892238 | 0.937 | AGAP029910 | protein coding gene - unspecified product |
|  | 7332239-7333238 | 0.937 | AGAP004970 | protein coding gene - unspecified product |
|  |  |  | AGAP004971 | protein coding gene - unspecified product |
|  | 7838239-7839238, 7873239-7874238 | 0.905 | AGAP005008 | protein coding gene - unspecified product |
|  | 9956239-9957238 | 0.902 | AGAP005093 | protein coding gene - unspecified product |
|  | 10159239-10160238 | 0.937 | AGAP005106 | protein coding gene - unspecified product |
|  | 10335239-10336238 | 0.937 | AGAP005127 | RNA-binding protein 15 |
|  | 12182239-12183238 | 0.937 | AGAP005197 | E2 ubiquitin-conjugating enzyme, bendless |
|  | 12220239-12221238 | 0.937 | AGAP005205 | peptidoglycan recognition protein (long) |
|  | 12448239-12449238 | 0.937 | AGAP005219 | protein coding gene - unspecified product |
|  | 12672239-12673238 | 0.905 | AGAP005235 | protein coding gene - unspecified product |
|  | 12999239-13000238 | 0.905 | AGAP005246 | serine protease inhibitor (serpin) 10 |
|  | 13330239-13331238 | 0.937 | AGAP029530 | protein coding gene - unspecified product |
|  | 13509239-13510238 | 0.937 | AGAP005273 | LRRCT domain-containing protein |
|  | 13842239-13843238 | 0.906 | AGAP005300 | protein coding gene - unspecified product |
|  | 14259239-14260238 | 0.937 | AGAP005339 | chitinase |
|  | 14448239-14449238 | 0.937 | AGAP005351 | DEAD box polypeptide 5 |
|  | 14643239-14644238 | 0.937 | AGAP005359 | F-box and WD-40 domain protein 7 |
|  | 14682239-14683238 | 0.937 | AGAP005368 | protein coding gene - unspecified product |
|  | 16464239-16465238 | 0.937 | AGAP005508 | protein coding gene - unspecified product |
|  | 16692239-16693238 | 0.905 | AGAP005534 | protein coding gene - unspecified product |
|  | 16962239-16963238 | 0.937 | AGAP005549 | Pre-mRNA-splicing factor rse1 |
|  | 16992239-16993238 | 0.937 | AGAP005553 | cellular retinaldehyde binding protein |
|  | 16997239-16998238 | 0.937 | AGAP005554 | protein coding gene - unspecified product |
|  | 20056239-20057238 | 0.937 | AGAP029489 | protein coding gene - unspecified product |
|  | 20304239-20305238 | 0.905 | AGAP005759 | DUF2236 domain-containing protein |
|  | 22662239-22663238 | 0.937 | AGAP005866 | glutamate decarboxylase |
|  | 24444239-24445238 | 0.905 | AGAP005978 | zinc finger and SCAN domain containing 2 |
|  |  |  | AGAP005979 | protein coding gene - unspecified product |
|  | 25392239-25393238 | 0.937 | AGAP006028 | GABA-gated chloride channel subunit |
|  | 25525239-25526238 | 0.937 | AGAP006038 | serine/arginine repetitive matrix protein 2 |
|  | 25592239-25593238 | 0.937 | AGAP006045 | protein yorkie |
|  | 25970239-25971238 | 0.905 | AGAP006063 | protein coding gene - unspecified product |
|  | 26504239-26505238 | 0.905 | AGAP006089 | protein coding gene - unspecified product |
|  | 30252239-30253238 | 0.905 | AGAP006347 | potassium voltage-gated channel KQT-like subfamily, invertebrate |
|  | 30424239-30425238 | 0.937 | AGAP006350 | defective proboscis extension response 11 |
|  | 32692239-32693238 | 0.902 | AGAP006475 | protein coding gene - unspecified product |
|  | 35674239-35675238 | 0.937 | AGAP006652 | ubiquitin carboxyl-terminal hydrolase 10 |
|  | 37076239-37077238 | 0.937 | AGAP006702 | protein coding gene - unspecified product |
|  | 38676239-38677238 | 0.937 | AGAP006805 | Lariat debranching enzyme |
|  | 40223239-40224238 | 0.905 | AGAP006954 | CLIP-domain serine protease |
|  | 45424239-45425238 | 0.937 | AGAP007327 | protein coding gene - unspecified product |
|  | 45781239-45782238 | 0.905 | AGAP028466 | protein coding gene - unspecified product |
|  | 46057239-46058238 | 0.905 | AGAP007361 | translocation protein SEC63 |
|  | 46122239-46123238 | 0.937 | AGAP007369 | Ras-related protein Rap-1b precursor |
|  | 46467239-46468238 | 0.905 | AGAP007416 | MH2 domain-containing protein |
|  | 46728239-46729238 | 0.937 | AGAP007465 | protein coding gene - unspecified product |
|  | 46949239-46950238 | 0.937 | AGAP007500 | Lachesin |
|  | 47187239-47188238 | 0.921 | AGAP007523 | myosin heavy chain |
|  | 47638239-47639238 | 0.905 | AGAP007563 | protein coding gene - unspecified product |
|  | 47756239-47757238 | 0.937 | AGAP007567 | membrane bound O-acyltransferase domain containing 2a |
|  | 47786239-47787238 | 0.937 | AGAP007573 | RAD54-like protein 2 |
|  | 48026239-48027238 | 0.937 | AGAP007587 | protein coding gene - unspecified product |
|  | 48549239-48550238 | 0.937 | AGAP007640 | protein coding gene - unspecified product |
|  | 48883239-48884238 | 0.937 | AGAP007667 | sugar transporter ERD6-like 4 |
|  | 49188239-49189238 | 0.937 | AGAP007712 | A-kinase anchor protein 13 |

Supplementary Table 2: Single Nucleotide Polymorphisms (SNPs) with F_ST_ ≥ 0.9 for chromosome 2R: comparison between An. melas from the Bijagós Archipelago with An. melas from Cameroon.

| **Chromosome** | **Window (Genomic bps)** | **F_ST_ Value** | **Gene ID** | **Protein coding gene** |
| --- | --- | --- | --- | --- |
| 2R | 644736-645735 | 0.905 | AGAP001157 | UBR-type domain-containing protein |
|  | 748736-749735 | 0.905 | AGAP001179 | nuclear pore complex protein Nup88 |
|  | 2467736-2468735 | 0.905 | AGAP012950 | protein coding gene - unspecified product |
|  | 5358736-5359735 | 0.905 | AGAP001472 | SAP domain-containing protein |
|  | 7212736-7213735 | 0.937 | AGAP001635 | sodium-coupled monocarboxylate transporter 2 |
|  | 8460736-8461735 | 0.937 | AGAP001690 | protein coding gene - unspecified product |
|  | 9205736-9206735 | 0.937 | AGAP001743 | estrogen-related receptor ERR |
|  | 10340736-10341735 | 0.905 | AGAP001786 | protein coding gene - unspecified product |
|  | 11033736-11034735 | 0.921 | AGAP001824 | protein coding gene - unspecified product |
|  | 11482736-11483735 | 0.937 | AGAP029451 | ATP-binding cassette transporter (ABC transporter) family G member 3 |
|  | 11983736-11984735 | 0.905 | AGAP001905 | zinc finger RNA-binding protein |
|  | 14010736-14011735 | 0.937 | AGAP002019 | COMPASS component SWD3 |
|  | 16660736-16661735 | 0.937 | AGAP029491 | protein coding gene - unspecified product |
|  | 16871736-16872735 | 0.937 | AGAP002172 | POU domain transcription factor, class 4 |
|  | 18039736-18040735 | 0.937 | AGAP002238 | protein coding gene - unspecified product |
|  | 18263736-18264735 | 0.906 | AGAP002261 | Ras GTPase-activating protein 3 |
|  | 19415736-19416735 | 0.937 | AGAP002315 | autophagy related gene |
|  | 19950736-19951735 | 0.937 | AGAP002329 | carnitine O-octanoyltransferase |
|  | 20116736-20117735 | 0.905 | AGAP002336 | Ig-like domain-containing protein |
|  | 22059736-22060735 | 0.937 | AGAP002503 | 4-coumarate:CoA ligase |
|  |  |  | AGAP002502 | translation initiation factor 4G |
|  | 22085736-22086735 | 0.937 | AGAP002505 | voltage-dependent calcium channel alpha 1, invertebrate |
|  | 23047736-23048735 | 0.905 | AGAP002573 | GTPase-activating Rap/Ran-GAP domain-like protein 3 |
|  | 23135736-23136735 | 0.905 | AGAP002577 | voltage-dependent p/q type calcium channel |
|  | 23177736-23178735 | 0.937 | AGAP002578 | protein coding gene - unspecified product |
|  | 23276736-23277735 | 0.903 | AGAP002581 | protein coding gene - unspecified product |
|  | 23291736-23292735 | 0.937 | AGAP002586 | glycogen(starch) synthase |
|  | 24200736-24201735 | 0.937 | AGAP012958 | IRF-2BP1_2 domain-containing protein |
|  | 25093736-25094735 | 0.905 | AGAP002649 | protein coding gene - unspecified product |
|  | 25186736-25187735 | 0.937 | AGAP002654 | protein coding gene - unspecified product |
|  | 25886736-25887735 | 0.937 | AGAP002707 | defective proboscis extension response 18 |
|  | 28401736-28402735 | 0.905 | AGAP002859 | solute carrier family 8 (sodium/calcium exchanger) |
|  | 29658736-29659735 | 0.937 | AGAP002924 | protein tweety homolog |
|  | 29838736-29839735 | 0.937 | AGAP002935 | Eukaryotic translation initiation factor 3 subunit F |
|  | 32937736-32938735 | 0.937 | AGAP003124 | dihydropyrimidinase |
|  | 33575736-33576735 | 0.905 | AGAP013292 | Rho-GAP domain-containing protein |
|  | 33590736-33591735 | 0.905 | AGAP003180 | protein coding gene - unspecified product |
|  |  |  | AGAP029060 | protein coding gene - unspecified product |
|  | 34787736-34788735 | 0.905 | AGAP003276 | splicing factor, arginine/serine-rich 17 |
|  | 34917736-34918735 | 0.937 | AGAP003283 | atrial natriuretic peptide receptor A |
|  | 35926736-35927735 | 0.937 | AGAP003318 | protein coding gene - unspecified product |
|  | 36885736-36886735 | 0.905 | AGAP003366 | myotubularin-related protein 13 |
|  | 37843736-37844735 | 0.937 | AGAP003445 | tuberous sclerosis 1 |
|  | 37869736-37870735 | 0.937 | AGAP003449 | rootletin |
|  | 37966736-37967735 | 0.937 | AGAP003463 | protein coding gene - unspecified product |
|  | 39069736-39070735 | 0.937 | AGAP003516 | protein coding gene - unspecified product |
|  | 40460736-40461735 | 0.905 | AGAP003604 | protein coding gene - unspecified product |
|  | 40512736-40513735 | 0.937 | AGAP003613 | palmitoyltransferase ZDHHC3 |
|  | 44341736-44342735 | 0.937 | AGAP003845 | bromodomain-containing protein 8 |
|  | 45584736-45585735 | 0.905 | AGAP003887 | Knickkopf |
|  | 46351736-46352735 | 0.905 | AGAP003925 | kinesin family member 1/13/14 |
|  | 47409736-47410735 | 0.905 | AGAP003976 | protein coding gene - unspecified product |
|  | 47718736-47719735 | 0.937 | AGAP003997 | casein kinase 1, gamma |
|  | 48605736-48606735, 48610736-48611735 | 0.905 | AGAP004046 | Mpv17-like protein |
|  | 49938736-49939735 | 0.905 | AGAP004106 | protein coding gene - unspecified product |
|  |  |  | AGAP004107 | protein coding gene - unspecified product |
|  | 50126736-50127735 | 0.937 | AGAP004112 | protein kinase domain-containing protein |
|  | 51445736-51446735 | 0.937 | AGAP004184 | protein coding gene - unspecified product |
|  | 51641736-51642735 | 0.937 | AGAP004192 | heat shock 70kDa protein 5 |
|  | 51964736-51965735 | 0.905 | AGAP004215 | protein coding gene - unspecified product |
|  | 53248736-53249735 | 0.905 | AGAP004249 | Methyltransf_11 domain-containing protein |
|  | 54363736-54364735 | 0.905 | AGAP004310 | protein coding gene - unspecified product |
|  | 54375736-54376735 | 0.937 | AGAP004311 | phosphopantothenate-cysteine ligase |
|  | 55236736-55237735 | 0.937 | AGAP004369 | protein coding gene - unspecified product |
|  | 55696736-55697735 | 0.905 | AGAP004402 | solute carrier family 17 member 7 |
|  | 56153736-56154735, 56178736 -56179735 | 0.919 | AGAP004443 | glycogen synthase kinase 3 beta |
|  | 56563736-56564735 | 0.937 | AGAP004453 | GPCR Dopamine Family 3 |
|  | 56740736-56741735 | 0.937 | AGAP013052 | protein coding gene - unspecified product |
|  |  |  | AGAP004474 | protein coding gene - unspecified product |
|  | 56805736-56806735 | 0.905 | AGAP004483 | Lethal giant larvae |

Supplementary Table 3: Single Nucleotide Polymorphisms (SNPs) with F^ST^ ≥ 0.9 for chromosome 3L: comparison between An. melas from the Bijagós Archipelago with An. melas from Cameroon.

| **Chromosome** | **Window (Genomic bps)** | **F_ST_ Value** | **Gene ID** | **Protein coding gene** |
| --- | --- | --- | --- | --- |
| 3L | 2813051-2814050 | 0.905 | AGAP010410 | protein coding gene - unspecified product |
|  | 3062051-3063050,  3064051-3065050 | 0.937 | AGAP010422 | Limbic system-associated membrane protein |
|  | 3316051-3317050 | 0.937 | AGAP010435 | protein coding gene - unspecified product |
|  | 3893051-3894050 | 0.916 | AGAP010456 | protein coding gene - unspecified product |
|  | 5011051-5012050 | 0.937 | AGAP010490 | rabconnectin |
|  | 6257051-6258050, 6259051-6260050 | 0.937 | AGAP010551 | STE20-like kinase |
|  | 7928051-7929050 | 0.921 | AGAP010642 | WH2 domain-containing protein |
|  | 7931051-7932050 | 0.937 | AGAP010643 | protein coding gene - unspecified product |
|  | 8613051-8614050 | 0.937 | AGAP028154 | V-type proton ATPase proteolipid subunit |
|  | 10390051-10391050 | 0.905 | AGAP029197 | Fatty acyl-CoA reductase |
|  | 12417051-12418050 | 0.937 | AGAP010883 | kinesin-like protein costa |
|  | 12418051-12419050 | 0.937 | AGAP010884 | protein coding gene - unspecified product |
|  | 12759051-12760050 | 0.937 | AGAP028617 | Sortilin-related receptor |
|  | 12896051-12897050 | 0.937 | AGAP010910 | phosphatidylinositol glycan, class Z |
|  | 13496051-13497050 | 0.905 | AGAP010939 | autophagy related gene |
|  | 16550051-16551050 | 0.905 | AGAP011094 | protein coding gene - unspecified product |
|  | 17204051-17205050 | 0.937 | AGAP011113 | potassium voltage-gated channel KQT-like subfamily member 1 |
|  | 20503051-20504050 | 0.905 | AGAP011279 | solute carrier family 6 (neurotransmitter transporter, amino acid/orphan) |
|  | 21595051-21596050 | 0.937 | AGAP011349 | GABA-gated chloride channel |
|  | 21754051-21755050 | 0.937 | AGAP011355 | protein coding gene - unspecified product |
|  | 23138051-23139050 | 0.905 | AGAP011384 | protein coding gene - unspecified product |
|  | 24403051-24404050 | 0.902 | AGAP011421 | IQ motif and SEC7 domain-containing protein |
|  | 24589051-24590050 | 0.905 | AGAP011433 | protein coding gene - unspecified product |
|  | 24760051-24761050 | 0.905 | AGAP011446 | far upstream element-binding protein |
|  | 25469051-25470050, 25660051-25661050 | 0.937 | AGAP029048 | Electroneutral potassium-chloride cotransporter 1 |
|  | 27994051-27995050 | 0.901 | AGAP011573 | protein coding gene - unspecified product |
|  | 29931051-29932050 | 0.937 | AGAP011618 | DOMON domain-containing protein |
|  | 31421051-31422050 | 0.905 | AGAP029516 | protein coding gene - unspecified product |
|  | 31497051-31498050 | 0.937 | AGAP011695 | homeobox protein SIX1 |
|  | 32456051-32457050 | 0.937 | AGAP029606 | protein coding gene - unspecified product |
|  | 33225051-33226050 | 0.937 | AGAP011779 | TAK1-associated binding protein 2 |
|  | 33453051-33454050 | 0.937 | AGAP011809 | protein coding gene - unspecified product |
|  | 33951051-33952050 | 0.906 | AGAP029637 | protein coding gene - unspecified product |
|  | 34665051-34666050 | 0.937 | AGAP011893 | Potentail helicase MOV-10 |
|  | 35229051-35230050 | 0.937 | AGAP011929 | protein coding gene - unspecified product |
|  | 36011051-36012050 | 0.937 | AGAP029590 | protein coding gene - unspecified product |
|  | 36050051-36051050 | 0.937 | AGAP012000 | fibrinogen and fibronectin |
|  | 36522051-36523050 | 0.905 | AGAP012023 | DBF4-type domain-containing protein |
|  | 36683051-36684050 | 0.916 | AGAP028133 | protein coding gene - unspecified product |
|  | 37106051-37107050 | 0.937 | AGAP029616 | RRM domain-containing protein |
|  | 37207051-37208050 | 0.937 | AGAP029469 | Non-specific serine/threonine protein kinase |
|  | 37403051-37404050 | 0.905 | AGAP029447 | protein coding gene - unspecified product |
|  | 37463051-37464050 | 0.902 | AGAP012079 | Miranda |
|  | 37486051-37487050 | 0.905 | AGAP029653 | protein coding gene - unspecified product |
|  | 37671051-37672050 | 0.937 | AGAP012103 | Insulin-like growth factor 2 mRNA-binding protein 2 |
|  | 37766051-37767050 | 0.937 | AGAP012113 | ubiquitin carboxyl-terminal hydrolase L3 |
|  | 37857051-37858050 | 0.937 | AGAP012123 | F-box and leucine-rich repeat protein 16 |
|  | 38092051-38093050 | 0.937 | AGAP012154 | solute carrier family 15 member 1 |
|  | 38570051-38571050 | 0.937 | AGAP012193 | ubiquitin-conjugating enzyme E2 F |
|  | 38574051-38575050 | 0.905 | AGAP012194 | DnaJ (Hsp40) homolog, subfamily B |
|  | 39339051-39340050 | 0.937 | AGAP012252 | classical protein kinase C |

Supplementary Table 4: Single Nucleotide Polymorphisms (SNPs) with F^ST^ ≥ 0.9 for chromosome 3R: comparison between An. melas from the Bijagós Archipelago with An. melas from Cameroon.

| **Chromosome** | **Window (Genomic bps)** | **F_ST_ Value** | **Gene ID** | **Protein coding gene** |
| --- | --- | --- | --- | --- |
| 3R | 528366-529365 | 0.937 | AGAP007771 | Nucleic_acid_bd domain-containing protein |
|  | 1245366-1246365,  1309366-1310365 | 0.937 | AGAP007803 | protein coding gene - unspecified product |
|  | 1543366-1544365 | 0.937 | AGAP007836 | sodium-coupled monocarboxylate transporter 2 |
|  | 1553366-1554365 | 0.921 | AGAP007838 | Sodium-coupled monocarboxylate transporter 2 |
|  | 2085366-2086365 | 0.905 | AGAP007849 | laminin, alpha 1/2 |
|  | 2420366-2421365 | 0.937 | AGAP007877 | protein coding gene - unspecified product |
|  | 2585366-2586365 | 0.937 | AGAP007901 | Ras-related protein Rab-5C |
|  | 2914366-2915365 | 0.905 | AGAP007925 | Ralgapb |
|  | 2939366-2940365 | 0.905 | AGAP007928 | Turtle protein, isoform |
|  | 3296366-3297365 | 0.905 | AGAP007955 | protein coding gene - unspecified product |
|  | 5111366-5112365 | 0.937 | AGAP008077 | protein coding gene - unspecified product |
|  | 8799366-8800365 | 0.905 | AGAP008304 | 3',5'-cyclic-nucleotide phosphodiesterase |
|  | 9612366-9613365 | 0.937 | AGAP008347 | Putative glycoprotein hormone rk-like receptor |
|  | 10075366-10076365 | 0.937 | AGAP008364 | thioester-containing protein 15 (TEP15) |
|  | 10214366-10215365 | 0.905 | AGAP008384 | protein coding gene - unspecified product |
|  | 10555366-10556365 | 0.937 | AGAP008418 | Rab-GAP TBC domain-containing protein |
|  | 13746366-13747365 | 0.937 | AGAP008626 | Androgen-induced 1 |
|  | 13834366-13835365 | 0.906 | AGAP008640 | protein coding gene - unspecified product |
|  | 13961366-13962365 | 0.905 | AGAP008646 | cGMP-specific 3',5'-cyclic phosphodiesterase |
|  | 15993366-15994365 | 0.937 | AGAP008717 | hydroxymethylglutaryl-CoA lyase |
|  | 18219366-18220365 | 0.937 | AGAP008814 | transcription factor 4/12 |
|  | 19492366-19493365 | 0.937 | AGAP028027 | protein coding gene - unspecified product |
|  | 20311366-20312365 | 0.905 | AGAP008889 | ATP-binding cassette transporter (ABC transporter) family G member 1 |
|  | 20661366-20662365 | 0.937 | AGAP008912 | Rac GTPase-activating protein 1 |
|  | 22484366-22485365 | 0.905 | AGAP008979 | protein coding gene - unspecified product |
|  | 25851366-25852365 | 0.937 | AGAP009112 | TPR_REGION domain-containing protein |
|  | 27722366-27723365 | 0.937 | AGAP009158 | protein coding gene - unspecified product |
|  | 31271366-31272365 | 0.937 | AGAP009325 | protein coding gene - unspecified product |
|  | 31800366-31801365 | 0.937 | AGAP009364 | protein coding gene - unspecified product |
|  | 32994366-32995365 | 0.905 | AGAP009424 | Calponin-homology (CH) domain-containing protein |
|  | 33476366-33477365 | 0.937 | AGAP009432 | Daxx-like protein |
|  | 34725366-34726365 | 0.925 | AGAP009493 | nicotinic acetylcholine receptor subunit alpha 9 |
|  | 35330366-35331365 | 0.937 | AGAP009522 | protein coding gene - unspecified product |
|  | 35605366-35606365 | 0.916 | AGAP009537 | cytochrome c |
|  | 36058366-36059365 | 0.937 | AGAP029897 | protein coding gene - unspecified product |
|  | 36546366-36547365 | 0.905 | AGAP009580 | protein coding gene - unspecified product |
|  | 37167366-37168365 | 0.905 | AGAP009625 | protein coding gene - unspecified product |
|  | 37243366-37244365 | 0.921 | AGAP009626 | Kv channel-interacting protein 4 isoform 2 |
|  | 37472366-37473365 | 0.937 | AGAP009641 | kelch-like protein diablo |
|  | 37663366-37664365 | 0.905 | AGAP029517 | protein coding gene - unspecified product |
|  | 41751366-41752365 | 0.937 | AGAP029589 | protein coding gene - unspecified product |
|  | 41865366-41866365 | 0.937 | AGAP029558 | Protein Wnt |
|  | 42190366-42191365 | 0.937 | AGAP028645 | Aryl hydrocarbon receptor nuclear translocator |
|  | 43190366-43191365, 43201366-43202365 | 0.937 | AGAP009777 | mothers against decapentaplegic homolog 2/3 |
|  | 43366366-43367365 | 0.905 | AGAP009793 | protein coding gene - unspecified product |
|  | 44349366-44350365 | 0.937 | AGAP009853 | gustatory receptor 5 |
|  | 44516366-44517365 | 0.937 | AGAP009860 | protein coding gene - unspecified product |
|  | 44741366-44742365 | 0.937 | AGAP029461 | protein coding gene - unspecified product |
|  | 45275366-45276365 | 0.937 | AGAP029826 | Tox-SGS domain-containing protein |
|  | 45444366-45445365 | 0.905 | AGAP009932 | protein coding gene - unspecified product |
|  | 46152366-46153365 | 0.905 | AGAP029448 | protein coding gene - unspecified product |
|  | 47386366-47387365 | 0.905 | AGAP009995 | anoctamin 10 |
|  | 47594366-47595365 | 0.905 | AGAP010012 | Leucine-rich repeat and calponin-like proteiny domain-containing protein 3 |
|  | 47851366-47852365 | 0.905 | AGAP029234 | protein coding gene - unspecified product |
|  | 49308366-49309365 | 0.937 | AGAP010135 | NCK adaptor protein |
|  | 50375366-50376365 | 0.903 | AGAP010189 | Arf-GAP domain-containing protein |

Supplementary Table 5: Single Nucleotide Polymorphisms (SNPs) with F^ST^ ≥ 0.9 for chromosome X: comparison between An. melas from the Bijagós Archipelago with An. melas from Cameroon.

| **Chromosome** | **Window (Genomic bps)** | **F_ST_ Value** | **Gene ID** | **Protein coding gene** |
| --- | --- | --- | --- | --- |
| X | 211895-212894 | 0.905 | AGAP000017 | protein coding gene - unspecified product |
|  | 447895-448894 | 0.937 | AGAP000035 | protein coding gene - unspecified product |
|  | 708895-709894 | 0.937 | AGAP000045 | G_PROTEIN_RECEP_F1_2 domain-containing protein |
|  | 1470895-1471894 | 0.905 | AGAP029109 | protein coding gene - unspecified product |
|  | 1744895-1745894 | 0.905 | AGAP000104 | protein coding gene - unspecified product |
|  | 1835895-1836894 | 0.937 | AGAP000112 | NAD+ synthase (glutamine-hydrolysing) |
|  | 2011895-2012894 | 0.937 | AGAP000123 | C-type lectin (CTL) |
|  | 2052895-2053894 | 0.905 | AGAP013373 | protein coding gene - unspecified product |
|  | 2185895-2186894 | 0.905 | AGAP000143 | beta-mannosidase |
|  | 2553895-  2555894 | 0.937 | AGAP013158 | protein coding gene - unspecified product |
|  | 2804895-2805894 | 0.937 | AGAP000159 | Kinesin-like protein |
|  | 2828895-2829894 | 0.937 | AGAP000161 | Adaptor-related protein complex 3, delta 1 subunit |
|  | 2910895-2911894 | 0.937 | AGAP000171 | E3 ubiquitin-protein ligase |
|  | 3421895-3422894 | 0.905 | AGAP000205 | protein coding gene - unspecified product |
|  | 4174895-4175894 | 0.905 | AGAP000223 | protein coding gene - unspecified product |
|  | 4231895-4232894 | 0.937 | AGAP000225 | protein coding gene - unspecified product |
|  | 4373895-4374894 | 0.937 | AGAP000232 | regulator of G-protein signaling |
|  | 4414895-4415894,  4420895-4421894 | 0.937 | AGAP000235 | Thymosin |
|  | 4436895-4437894 | 0.905 | AGAP000236 | 3',5'-cyclic-nucleotide phosphodiesterase |
|  | 4555895-4556894 | 0.937 | AGAP000243 | Rhophilin-2 |
|  | 4937895-4938894 | 0.905 | AGAP000264 | protein coding gene - unspecified product |
|  | 5318895-5319894 | 0.905 | AGAP000297 | protein coding gene - unspecified product |
|  | 5808895-5809894 | 0.937 | AGAP000331 | low density lipoprotein-related protein 2 |
|  | 6273895-6274894 | 0.937 | AGAP000351 | neuropeptide Y receptor 1 |
|  | 6988895-6989894 | 0.905 | AGAP000383 | protein coding gene - unspecified product |
|  | 7196895-7197894 | 0.905 | AGAP000393 | Trimeric intracellular cation channel type B |
|  | 7206895-7207894 | 0.937 | AGAP013009 | protein coding gene - unspecified product |
|  | 7250895-7251894 | 0.937 | AGAP000399 | squid |
|  | 7338895-7339894 | 0.937 | AGAP000410 | histone deacetylase 4/5 |
|  | 7560895-7561894 | 0.905 | AGAP000421 | protein coding gene - unspecified product |
|  | 7881895-7882894 | 0.937 | AGAP000449 | protein coding gene - unspecified product |
|  | 8295895-8296894,  8335895-8336894 | 0.937 | AGAP000477 | serine/threonine kinase 32 |
|  | 8696895-8697894 | 0.937 | AGAP029670 | protein coding gene - unspecified product |
|  | 9252895-9253894 | 0.937 | AGAP000519 | diacylglycerol kinase (ATP dependent) |
|  | 9414895-9415894,  9430895-9431894 | 0.905 | AGAP000522 | Ig-like domain-containing protein |
|  | 9717895-9718894 | 0.937 | AGAP013055 | protein coding gene - unspecified product |
|  | 10571895-10582894 | 0.905 | AGAP000591 | decaprenyl-diphosphate synthase subunit 1 |
|  | 10663895-10664894 | 0.919 | AGAP000596 | lysophosphatidylcholine acyltransferase / lyso-PAF acetyltransferase |
|  | 10692895-10693894 | 0.937 | AGAP000601 | LRRNT domain-containing protein |
|  | 11035895-11036894,  11117895-11118894 | 0.905 | AGAP000606 | putative GPCR class a orphan receptor 19 |
|  | 11236895-11237894 | 0.905 | AGAP000620 | protein coding gene - unspecified product |
|  | 11788895-11789894 | 0.937 | AGAP000663 | protein coding gene - unspecified product |
|  | 12170895-12171894 | 0.905 | AGAP000685 | protein coding gene - unspecified product |
|  | 14279895-14280894 | 0.912 | AGAP000785 | Synaptic vesicle protein |
|  | 14327895-14328894 | 0.937 | AGAP000787 | protein coding gene - unspecified product |
|  | 15416895-15417894 | 0.905 | AGAP000834 | protein coding gene - unspecified product |
|  | 16030895-16031894 | 0.905 | AGAP000863 | Lachesin |
|  | 16434895-16435894 | 0.937 | AGAP000874 | protein coding gene - unspecified product |
|  | 17022895-17023894 | 0.937 | AGAP000900 | Protein-tyrosine sulfotransferase |
|  | 17087895-17088894 | 0.905 | AGAP029488 | protein coding gene - unspecified product |
|  | 17455895-17456894 | 0.926 | AGAP000926 | inositol hexakisphosphate/diphosphoinositol-pentakisphosphate kinase |
|  | 17615895-17616894 | 0.905 | AGAP000932 | protein coding gene - unspecified product |
|  | 17823895-17824894 | 0.937 | AGAP029105 | protein coding gene - unspecified product |
|  | 18750895-18751894 | 0.937 | AGAP000974 | protein coding gene - unspecified product |
|  | 20011895-20012894 | 0.937 | AGAP001039 | cytochrome P450 – CYP307A1 |

Supplementary Table 6: Single Nucleotide Polymorphisms (SNPs) with F_ST_ ≥ 0.6 for the mitochondrial genome: comparison between An. melas from the Bijagós Archipelago with An. melas from Cameroon.

| **Chromosome** | **Window (Genomic bps)** | **F_ST_ Value** | **Gene ID** | **Protein coding gene** |
| --- | --- | --- | --- | --- |
| Mt | 1172-2171 | 0.661 | AGAP028360 | NADH dehydrogenase subunit 2 |
|  |  |  | AGAP028361 | Protein coding gene - unspecified product |
|  |  |  | AGAP028362 | Protein coding gene - unspecified product |
|  |  |  | AGAP028363 | Protein coding gene - unspecified product |
|  |  |  | AGAP028364 | cytochrome c oxidase subunit I |
|  | 5172-6171 | 0.750 | AGAP028371 | cytochrome c oxidase subunit III |
|  |  |  | AGAP028372 | tRNA-Gly for anticodon UCC |
|  |  |  | AGAP028373 | NADH dehydrogenase subunit 3 |
|  |  |  | AGAP028374 | Protein coding gene - unspecified product |
|  |  |  | AGAP028375 | Protein coding gene - unspecified product |
|  |  |  | AGAP028376 | Protein coding gene - unspecified product |
|  |  |  | AGAP028377 | Protein coding gene - unspecified product |
|  | 7172-8171 | 0.856 | AGAP028380 | NADH dehydrogenase subunit 5 |
|  |  |  | AGAP028381 | Protein coding gene - unspecified product |
|  |  |  | AGAP028382 | NADH dehydrogenase subunit 4 |

Supplementary Table 7: Non-synonymous SNPs associated/putatively associated with insecticide resistance which were investigated in the An. melas population

| **Gene** | **Chromosome** | **Average depth of coverage** | **Position** | **SNP** | **Frequency of SNP in *Anopheles melas*** |
| --- | --- | --- | --- | --- | --- |
| *vgsc* | 2L | 24.98 | 2390177 | R254K | 0% |
|  |  |  | 2391228 | V402L | 0% |
|  |  |  | 2399997 | D466H | 0% |
|  |  |  | **2400071** | **M490I** | **2%** |
|  |  |  | 2402466 | G531V | 0% |
|  |  |  | 2407967 | Q697P | 0% |
|  |  |  | 2416980 | T791M | 0% |
|  |  |  | 2422651 | L995S | 0% |
|  |  |  | 2422652 | L995F | 0% |
|  |  |  | 2429556 | V1507I | 0% |
|  |  |  | 2429617 | I1527T | 0% |
|  |  |  | 2429745 | N1570Y | 0% |
|  |  |  | 2429897 | E1597G | 0% |
|  |  |  | 2429915 | K1603T | 0% |
|  |  |  | 2430424 | A1746S | 0% |
|  |  |  | 2430817 | V1853I | 0% |
|  |  |  | 2430863 | I1868T | 0% |
|  |  |  | 2430880 | P1874S | 0% |
|  |  |  | 2430881 | P1874L | 0% |
|  |  |  | 2431061 | A1934V | 0% |
|  |  |  | 2431079 | I1940T | 0% |
| *gste2* | 3R | 22.21 | 28598166 | I114T | 0% |
|  |  |  | 28598057 | F120L | 0% |
|  |  |  | 28598062 | L119V | 0% |
| *rdl* | 2L | 19.70 | 25429236 | A296G | 0% |
|  |  |  | 25429235 | A296S | 0% |
| *ace1* | 2R | 21.45 | 3492074 | G280S* | 0% |


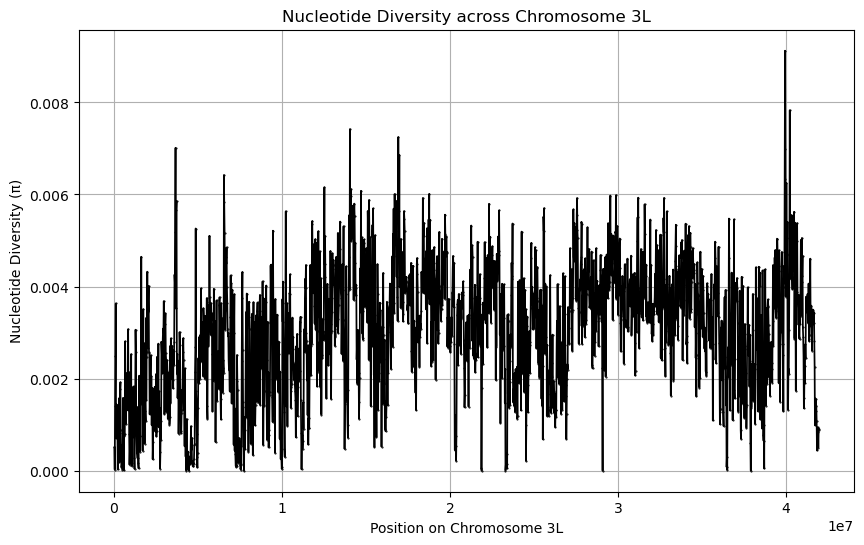


Supplementary Figure 2: Nucleotide diversity across chromosome 3L, Bijagós An. melas mosquitoes, N = 30


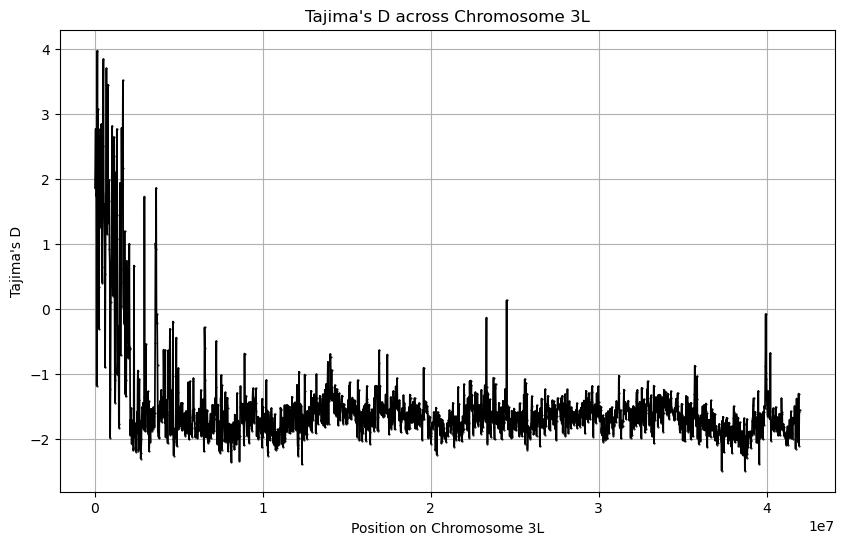


Supplementary Figure 3: Tajima's D computed across chromosome 3L, Bijagós An. melas mosquitoes, N = 30


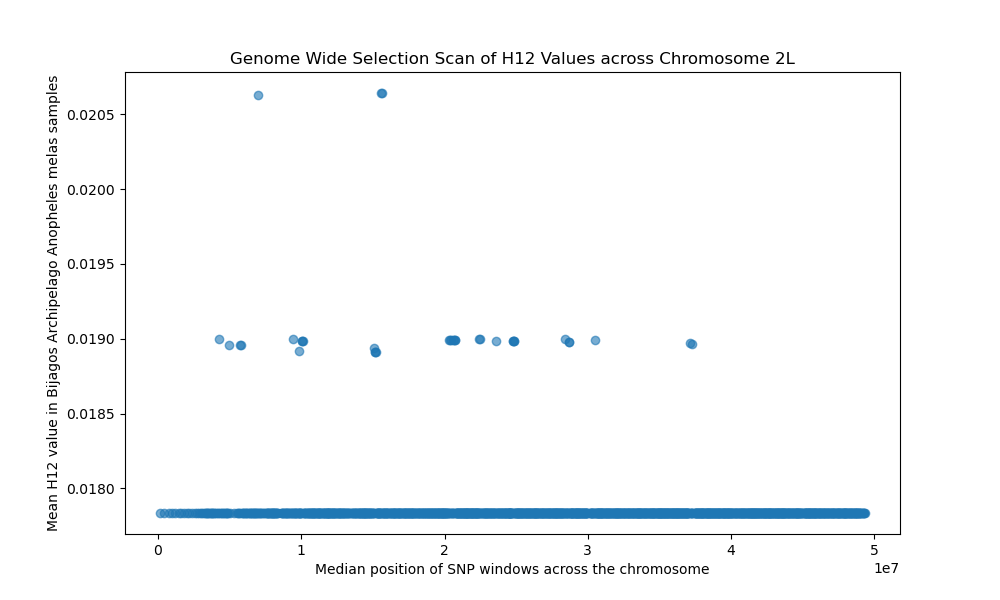


Supplementary Figure 4: Genome wide selection scan of H12 values across Chromosome 2L, Bijagós An. melas, N=30


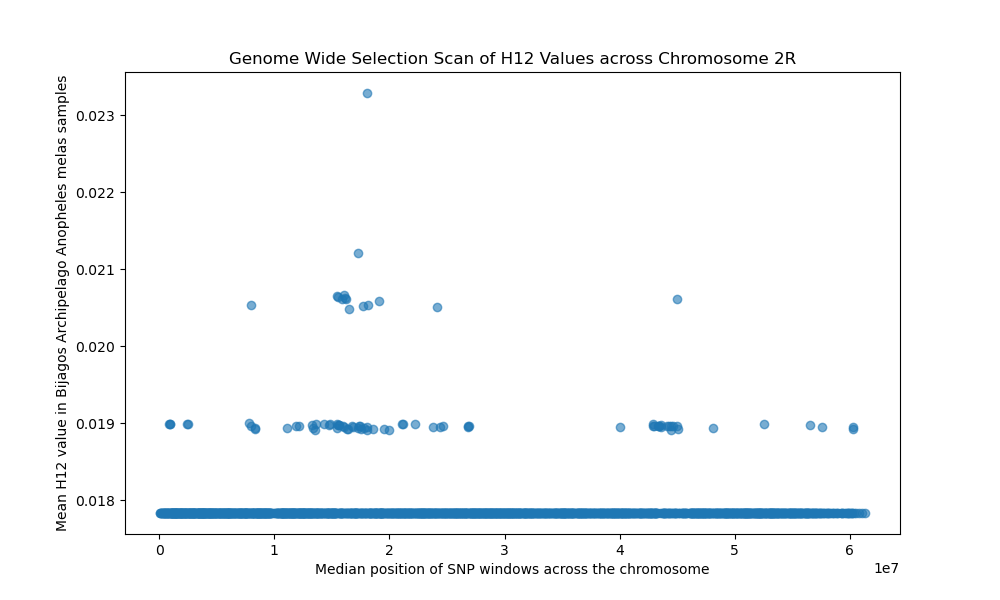


Supplementary Figure 5: Genome wide selection scan of H12 values across Chromosome 2R, Bijagós An. melas, N=30


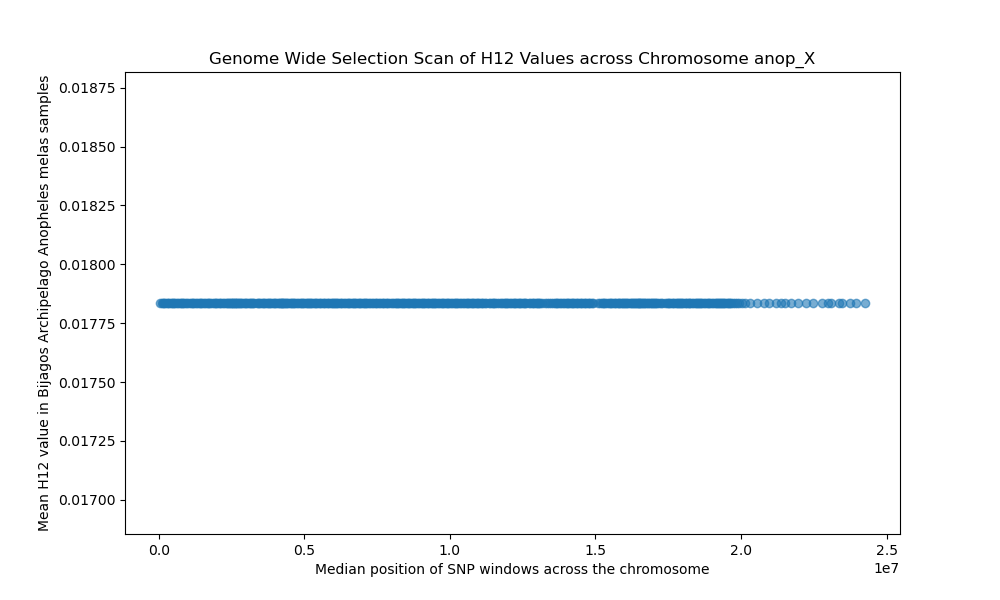


Supplementary Figure 6: Genome wide selection scan of H12 values across Chromosome X, Bijagós An. melas, N=30


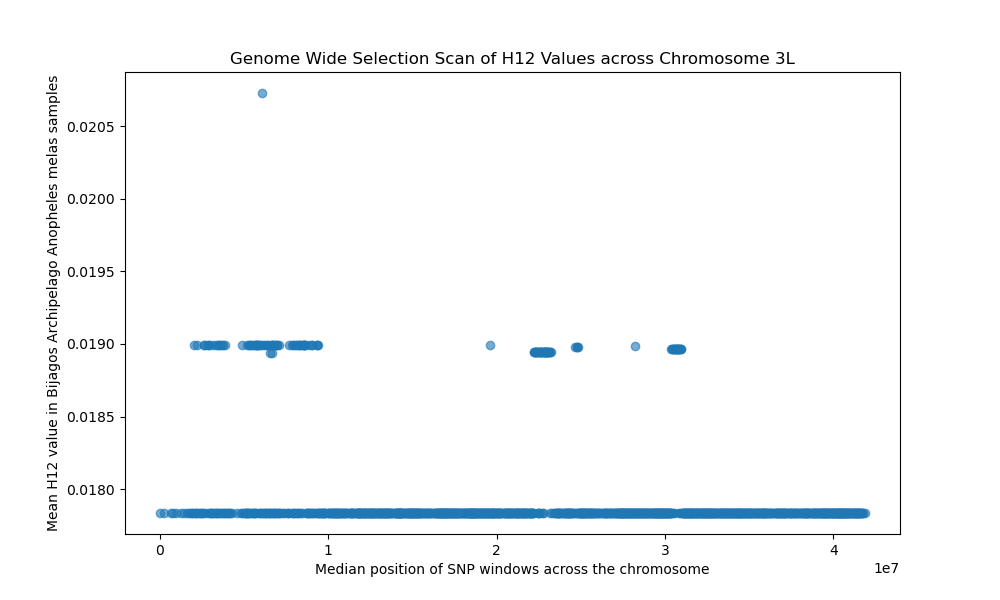


Supplementary Figure 7: Genome wide selection scan of H12 values across Chromosome 3L, Bijagós An. melas, N=30


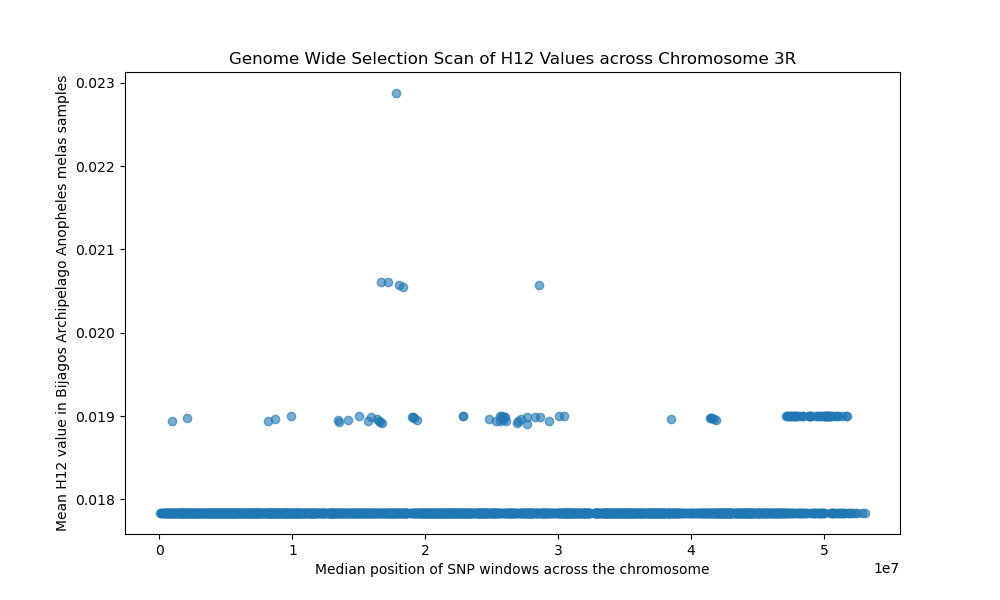


Supplementary Figure 8: Genome wide selection scan of H12 values across Chromosome 2R, Bijagós An. melas, N=30


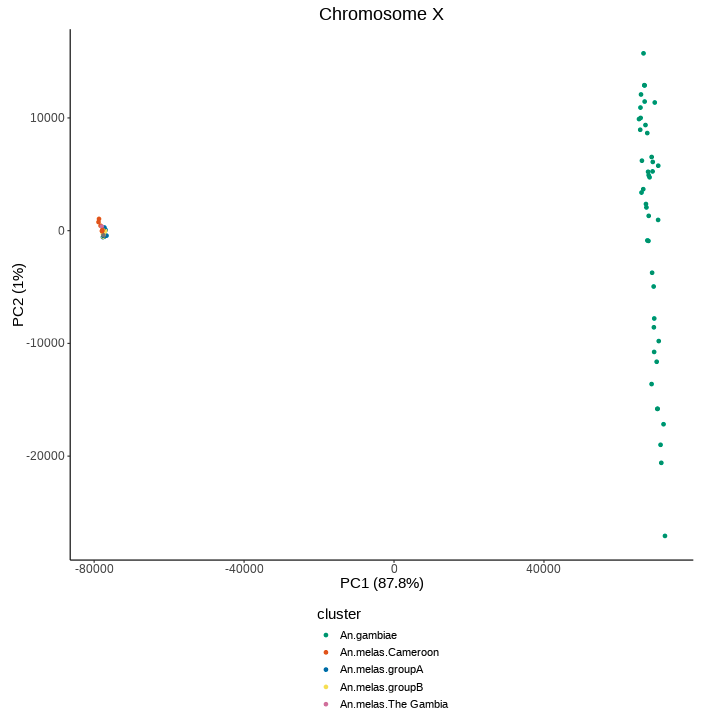


Supplementary Figure 9: Principal Components Analysis of chromosome X comparing An. melas and An. gambiae s.s. from the Bijagós Archipelago. Includes additional An. melas samples from Cameroon and The Gambia. An. gambiae and An. melas cluster separately.


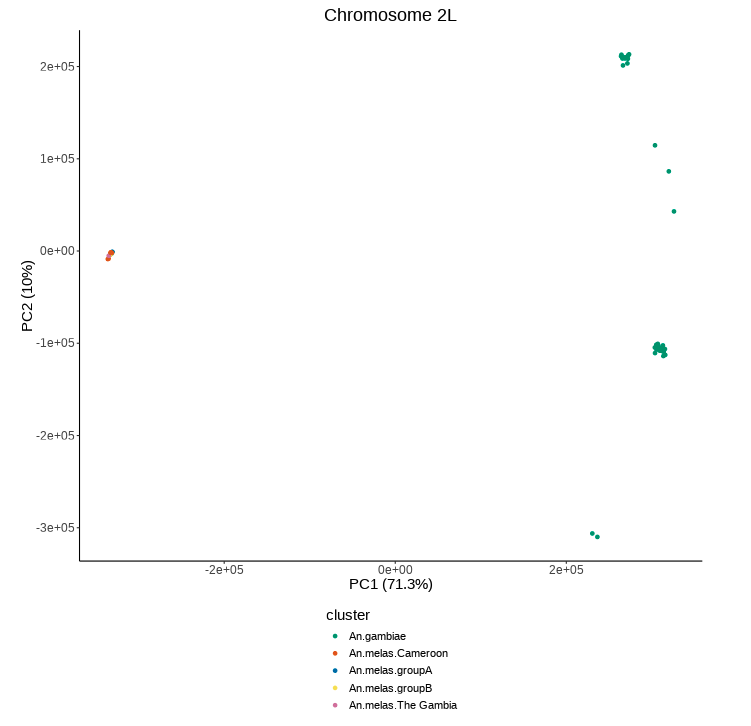


Supplementary Figure 10: Principal Components Analysis of chromosome 2L comparing An. melas and An. gambiae s.s. from the Bijagós Archipelago. Includes additional An. melas samples from Cameroon and The Gambia. An. gambiae and An. melas cluster separately.


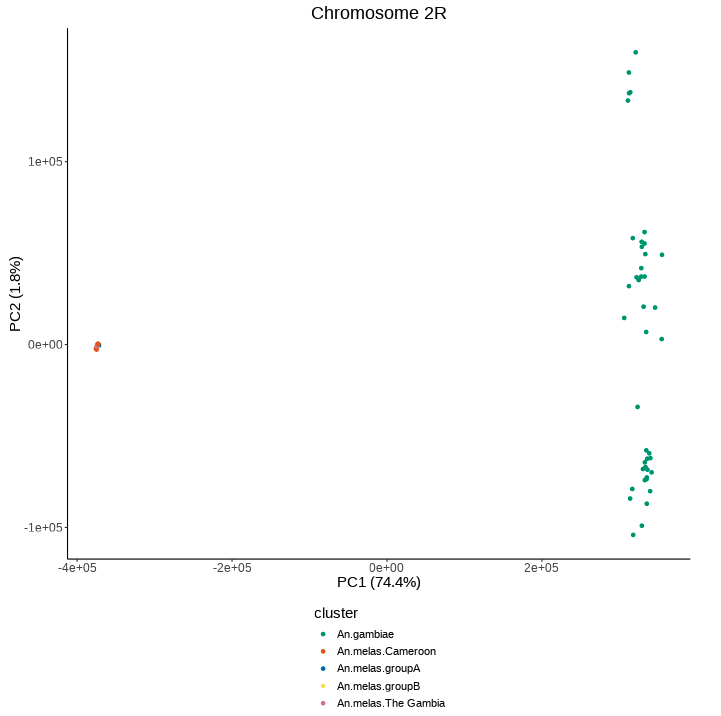


Supplementary Figure 11: Principal Components Analysis of chromosome 2R comparing An. melas and An. gambiae s.s. from the Bijagós Archipelago. Includes additional An. melas samples from Cameroon and The Gambia. An. gambiae and An. melas cluster separately.


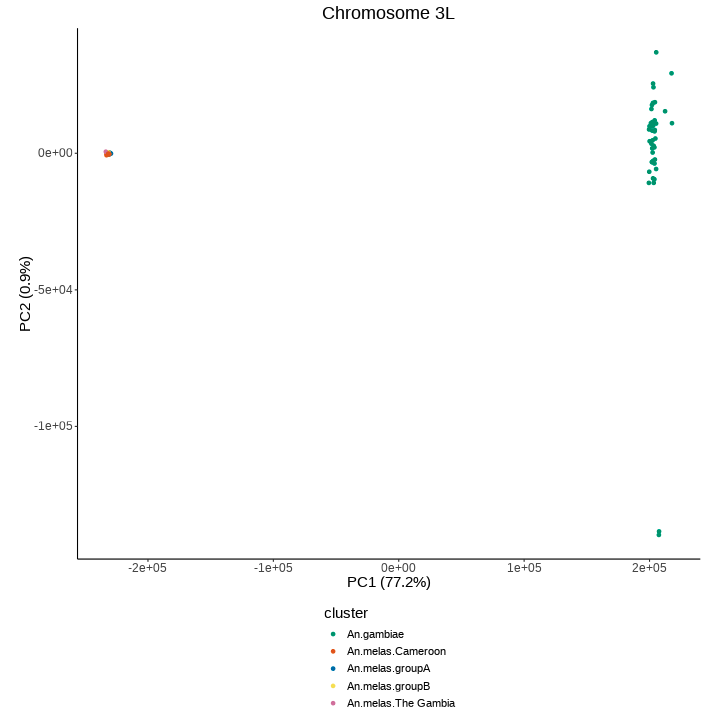


Supplementary Figure 12: Principal Components Analysis of chromosome 3L comparing An. melas and An. gambiae s.s. from the Bijagós Archipelago. Includes additional An. melas samples from Cameroon and The Gambia. An. gambiae and An. melas cluster separately.


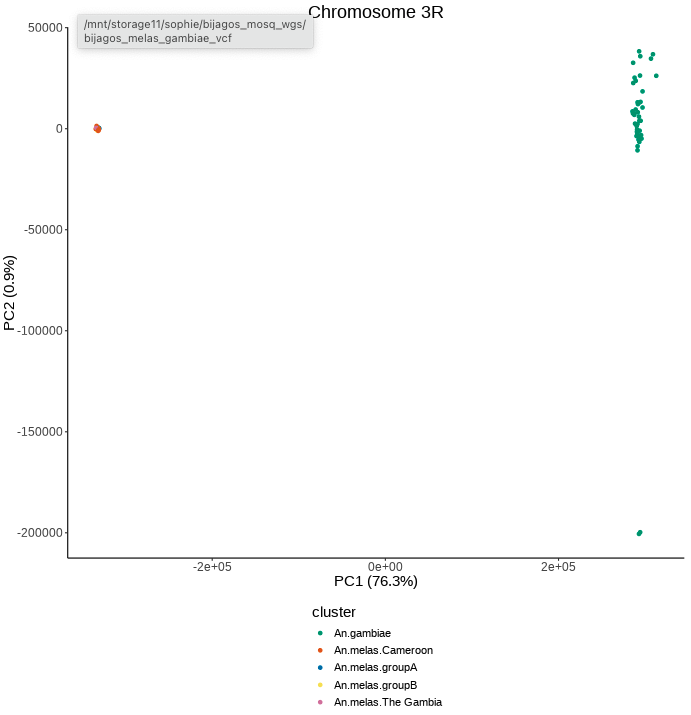


Supplementary Figure 13: Principal Components Analysis of chromosome 3R comparing An. melas and An. gambiae s.s. from the Bijagós Archipelago. Includes additional An. melas samples from Cameroon and The Gambia. An. gambiae and An. melas cluster separately.
